# Computational drug prediction in hepatoblastoma by integrating pan-cancer transcriptomics with pharmacological response

**DOI:** 10.1097/HEP.0000000000000601

**Published:** 2023-09-20

**Authors:** Mario Failli, Salih Demir, Álvaro Del Río-Álvarez, Juan Carrillo-Reixach, Laura Royo, Montserrat Domingo-Sàbat, Margaret Childs, Rudolf Maibach, Rita Alaggio, Piotr Czauderna, Bruce Morland, Sophie Branchereau, Stefano Cairo, Roland Kappler, Carolina Armengol, Diego di Bernardo

**Affiliations:** 1 Telethon Institute of Genetics and Medicine, Pozzuoli, Naples, Italy; 2 Department of Chemical, Materials and Industrial Production Engineering, University of Naples “Federico II”, Naples, Italy; 3 Department of Pediatric Surgery, Dr. von Hauner Children’s Hospital, University Hospital, LMU Munich, Germany; 4 Childhood Liver Oncology Group (c-LOG), Health Sciences Research Institute Germans Trias i Pujol (IGTP), Badalona, Catalonia, Spain; 5 Nottingham Clinical Trials Unit, Nottingham, United Kingdom; 6 International Breast Cancer Study Group Coordinating Center, Bern, Switzerland; 7 Pathology Unit, Bambino Gesù Children’s Hospital, IRCCS, Rome, Italy; 8 Department of Surgery and Urology for Children and Adolescents, Medical University of Gdansk, Gdansk, Poland; 9 Department of Oncology, Birmingham Women’s and Children’s Hospital, Birmingham, United Kingdom; 10 Bicêtre Hospital, Le Kremlin-Bicêtre, France; 11 XenTech, Evry, France; 12 Champions Oncology, Rockville, Maryland, USA; 13 Liver and Digestive Diseases Networking Biomedical Research Centre (CIBEREHD), Madrid, Spain

## Abstract

**Background and Aims:**

Hepatoblastoma (HB) is the predominant form of pediatric liver cancer, though it remains exceptionally rare. While treatment outcomes for children with HB have improved, patients with advanced tumors face limited therapeutic choices. Additionally, survivors often suffer from long-term adverse effects due to treatment, including ototoxicity, cardiotoxicity, delayed growth, and secondary tumors. Consequently, there is a pressing need to identify new and effective therapeutic strategies for patients with HB. Computational methods to predict drug sensitivity from a tumor's transcriptome have been successfully applied for some common adult malignancies, but specific efforts in pediatric cancers are lacking because of the paucity of data.

**Approach and Results:**

In this study, we used DrugSense to assess drug efficacy in patients with HB, particularly those with the aggressive C2 subtype associated with poor clinical outcomes. Our method relied on publicly available collections of pan-cancer transcriptional profiles and drug responses across 36 tumor types and 495 compounds. The drugs predicted to be most effective were experimentally validated using patient-derived xenograft models of HB grown in vitro and in vivo. We thus identified 2 cyclin-dependent kinase 9 inhibitors, alvocidib and dinaciclib as potent HB growth inhibitors for the high-risk C2 molecular subtype. We also found that in a cohort of 46 patients with HB, high cyclin-dependent kinase 9 tumor expression was significantly associated with poor prognosis.

**Conclusions:**

Our work proves the usefulness of computational methods trained on pan-cancer data sets to reposition drugs in rare pediatric cancers such as HB, and to help clinicians in choosing the best treatment options for their patients.

## Graphical abstract

**Figure:**
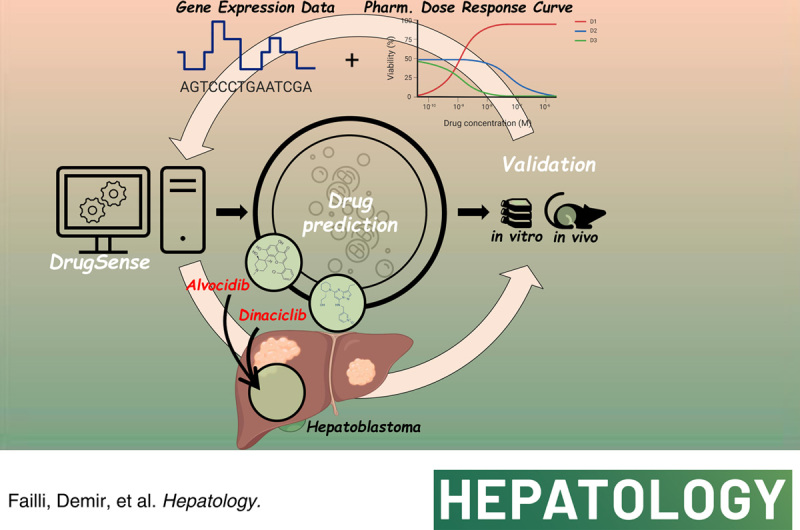

## INTRODUCTION

Hepatoblastoma (HB) is the main pediatric liver cancer; however, it is a very rare disease with an approximate incidence of one case in 1 million children per year.^1^ HB is characterized by a low mutation burden,^2^ but with a high recurrence of activating CTNNB1 mutations^3,4^; hence, the determinants of the clinical heterogeneity of HB are mainly related to differences in their transcriptome and epigenome rather than its genome. In line with this observation, 2 main transcriptomic (C1 and C2) and epigenomic (Epi-CA and Epi-CB) HB subtypes have been identified and associated with clinical behavior.^5,6^ In particular, the C2 subtype displays features of aggressive tumors characterized by having a stemness-like profile, high proliferation, and upregulation of MYC protein (MYC) target genes, resulting in a poor clinical outcome.

Significant improvements have occurred in the treatment of children diagnosed with HB. However, there are still limited treatment options for patients resistant to current treatments.^7^ Accordingly, there is an urgent need to define new and efficient therapeutic strategies for patients with HB.

Transcriptomic profiles have been effectively used to identify tumor subtypes, tumor-specific master regulators, and risk stratification in the most commonly occurring cancers.^8^ Moreover, computational methods to predict drug sensitivity from baseline molecular features of cancer cell lines (CCLs) have been described in the literature, aided by the availability of large-scale genomic and transcriptomic data together with cell line–specific response to hundreds of drugs and research compounds.^9–15^ Specifically, it has been shown that the basal gene expression profile (GEP) of a CCL, measured in bulk or even at the single-cell level, can be used to predict its sensitivity, or resistance, to hundreds of drugs^13,14,16,17^ and drug combinations.^18^

Here, we asked whether computational methods to predict drug sensitivity that are trained on pan-cancer transcriptional data sets could be successfully used to find therapeutic drugs also in the case of patients with HB, for which transcriptional profiles^5^ measured in the 2 prognostic C1 and C2 HB subtypes are available.

To this end, we implemented a simple computational approach, which we named DrugSense, to identify drugs specific for the C2 subtype starting from their tumor transcriptional profile. DrugSense exploits large publicly available data sets^9^ containing transcriptional profiles of 1375 pan-CCLs and their drug response to 495 compounds to identify gene expression biomarkers of both drug sensitivity and drug resistance for each compound. DrugSense then uses these biomarkers to identify the drugs to which the patient will be most sensitive to, starting from the tumor’s transcriptional profile.

We applied DrugSense to predict drug sensitivity in a cohort of 24 patients with HB. We found 2 cyclin-dependent kinase 9 (CDK9) inhibitors, alvocidib and dinaciclib, acting as potent HB growth inhibitors for the high-risk C2 tumor subtype,^5,6^ which were then further validated in HB patient-derived xenograft (PDX) models grown *in vitro* and *in vivo*.

### Computational prediction of drug response from GEPs

**FIGURE 1:**
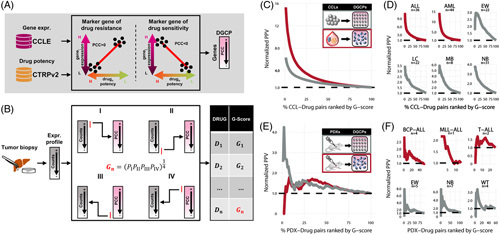
Overview of DrugSense and in silico validation in CCLs and PDX models in the Childhood Cancer Therapeutics portal. (A) Training phase: expression biomarkers of resistance and sensitivity are identified for each drug present in the CTRPv2 database. Biomarkers are selected by correlating gene expression with drug potency across cell lines. The drug potency is as the AUC of the dose-growth inhibition curve. Each dot refers to one cell line and indicates the cell line-specific expression of the gene (*y*-axis) and the cell line-specific drug potency (*x*-axis). The PCC between gene expression and drug potency is thus computed for each gene. Genes are ordered by PCC to obtain the DGCP. (B) Analysis of a tumor transcriptional profile: the gene expression profile is sorted from the most to the least expressed gene and compared against the DGCP of a drug by Gene Set Enrichment Analysis. The drug G-score is defined as the geometric mean of four *p*-values computed by performing 4 Gene Set Enrichment analyses to check whether: (1) the 250 most expressed genes in the tumor tend to be biomarkers of sensitivity; (2) the 250 least expressed genes in the tumor tend to be biomarkers of resistance; (3) the 250 biomarkers of sensitivity at the bottom of the DGCP tend to be upregulated in the tumor; (4) the 250 biomarkers of resistance at the top of the DGCP tend to be downregulated in the tumor. Drugs are then ranked by their G-score in ascending order according to their predicted potency. (C, D) CCL/drug pairs are ranked according to their G-score in ascending order and reported as percentiles on the *x*-axis. The PPV is normalized against the random PPV (black dashed line) obtained by ordering drug randomly rather than according to their G-score. Normalized PPVs for 672 CCLs from solid (gray) and 83 from liquid (red) tumors in (C), whereas PPV curves for 6 distinct collections of cell lines from pediatric tumors are shown in (D). (E, F) PDX/drug pairs are ranked according to their G-score in ascending order and reported as percentiles on the *x*-axis. Normalized PPV of DrugSense for all PDXs derived from solid tumors (gray) and liquid tumors (red) are shown in (E). PPV curves for PDX belonging to the 6 distinct pediatric tumors are shown in (F). Abbreviations: ALL, acute lymphocytic leukemia; AML, acute myeloid leukemia; BCP-ALL, B-cell precursor acute lymphoblastic leukemia; CCL, cancer cell line; CCLE, Cancer Cell Line Encyclopaedia; CTRPv2, Cancer Therapeutics Response Portal; DGCP, drug-gene correlation profile; EW, Ewing sarcoma; LC, liver cancer; MB, medulloblastoma; NB, neuroblastoma; MLL-ALL, MLL-rearranged acute lymphoblastic leukemia; PCC, Pearson correlation coefficient; PDX, patient-derived xenograft; T-ALL, T-cell acute lymphoblastic leukemia; WT, Wilms tumor.

DrugSense is schematically depicted in Figure 1A, B and it is divided into a training phase and an analysis phase. During the training phase, which is executed only once, DrugSense identifies biomarkers of sensitivity and resistance for each compound of interest by combining large-scale publicly available data sets of bulk GEPs of untreated cells from the Cancer Cell Line Encyclopaedia,^9^ with drug response data from the Cancer Therapeutics Response Portal (CTRPv2). These databases contain the bulk expression profiles of 1375 CCLs across 36 tumor types and their drug response to 495 compounds. Once these biomarkers have been identified during the analysis phase, they can be used to analyze the bulk GEP of a tumor and to score the drugs according to their predicted potency, as depicted in Figure 1B.

During the training phase, for each gene and for each drug (ie, a gene/drug pair), DrugSense computes the Pearson correlation coefficient between the expression of the gene and the *in vitro* response to the drug across all CCLs. As shown in Figure 1A, biomarkers of drug resistance must be positively correlated with the AUC of the dose-growth inhibition curve across CCLs (the more expressed the gene is in a CCL, the higher the drug concentration needed to inhibit cell growth); vice versa, a negative correlation denotes a biomarker of drug sensitivity. Genes are thus ranked according to their correlation coefficient to give rise to a “drug-gene correlation profile” (DGCP). Genes at the top of the DGCP are positively correlated with the AUC and are thus likely markers of resistance, and vice versa those at the bottom of the DGCP are likely markers of sensitivity. At the end of the training phase, for each of the 495 drugs in the CTRPv2 database, there will be a corresponding DCGP. In the analysis phase, DrugSense uses DGCPs to rank drugs according to their anticancer effect starting from the GEP of a tumor sample, as shown in Figure 1B. To do so, DrugSense performs four different gene set enrichment analysis (GSEA)^19^ for each drug. The geometric mean of individual probabilities (ie, the *G-score*) is then computed for the drug (Figure 1B). The smaller the *G*-score, the higher the likelihood that the tumor is sensitive to the drug. Finally, drugs are ranked in ascending order according to their *G*-score, with drugs with the smallest *G*-score at the top of the list.

DrugSense was trained separately on the 672 CCLs from solid tumors, for which drug potency data were available for 445 drugs, and on the 83 CCLs from liquid tumors across 414 drugs, resulting in a total of 859 (=445+414) DGCPs (Supplemental Figure S1A, B, http://links.lww.com/HEP/I17). Moreover, as shown in Supplemental Figure S1C, http://links.lww.com/HEP/I17 for both solid and liquid tumors, genes in the same pathways as the drug target tend to be significantly anticorrelated with the drug AUC as compared to genes belonging to pathways unrelated to the drug target (Mann-Whitney test *p*-value<2.2e-16), thus confirming the biological relevance of the resulting DGCPs.

To assess whether DrugSense could indeed predict drug sensitivity from a tumor’s transcriptional profiles, we first applied it to the GEPs of the very same CCLs used in the training phase. As a gold standard, we took advantage of the CTRPv2 database to assign to each CCL, one or more drugs to which the CCL is sensitive to, according to the reported experimental dose-response curves (Methods). As shown in Figure 1C, DrugSense precision for both solid and liquid CCLs proved to be up to 6-fold and 15-fold better than random, respectively. Figure 1D reports the performance in terms of positive predictive value (PPV) only for the subset of CCLs derived from pediatric tumors. As a negative control, we analyzed solid CCLs with DrugSense trained on liquid CCLs, and vice versa. In this case, the PPV is close to the random value, as expected (Supplemental Figure S1D, http://links.lww.com/HEP/I17). We also confirmed the robustness of DrugSense against algorithm’s parameters, specifically we varied the gene set size used for the GSEA analyses (Supplemental Figure S2, http://links.lww.com/HEP/I17) and also how individual -values are integrated when computing the G-score (Supplemental Figures S2 and S3, http://links.lww.com/HEP/I17). In all the tested cases, the overall PPV of DrugSense was only minimally affected.

As cell lines are more proliferative than tumors in a patient, the use of cell line–based drug-sensitivity profiles may bias toward the selection of antiproliferative agents that interfere with the cell cycle. Hence, we performed an additional analysis by first grouping drugs according to their known targets, so that drugs whose targets belong to the same biological pathways are in the same group. To this end, we extracted gene sets representing well-defined biological pathways from the manually curated Molecular Signature Database (MSigDB).^20^ We then calculated the PPVs for each group of drugs to assess for differences in predictive power. As shown in Supplemental Figure S4, http://links.lww.com/HEP/I17, we did not observe any bias for any biological pathway, including the cell cycle.

As an additional validation, we applied DrugSense to a separate data set of transcriptional profiles measured in the same CCLs but derived from the Genomics of Drug Sensitivity in Cancer database.^10^ The aim of the new benchmark was to assess whether applying DrugSense on transcriptional profiles not used during the training phase and measured with a different platform (ie, microarrays) would result in similar precision. PPV curves shown in Supplemental Figure S1E–G, http://links.lww.com/HEP/I17 confirm that this is the case.

We also assessed DrugSense performance in analyzing transcriptional profiles from a collection of 39 PDX for Childhood Cancer Therapeutics (PCAT) portal,^21^ of which 31 from solid tumors and 8 from liquid tumors, whose response to 44 drugs was experimentally measured. Out of these 44 drugs, 23 were present in DrugSense for solid tumors, and 21 for liquid tumors (Supplemental Figure S1H, I, http://links.lww.com/HEP/I17). We generated a gold standard by calling a PDX sensitive to a drug if it was classified in PCAT as inducing either a complete response or a partial response; if instead the PCAT classification was stable disease or progressive disease, the PDX was deemed resistant to the drug. DrugSense precision is reported in Figure 1E, F by plotting the PPV for all the PDX-drug pairs ranked in ascending order by their *G*-score.

DrugSense performed significantly better on solid tumor than liquid tumors; this could be due to the small sample size, as only 8 PDX models were available for liquid tumors versus 31 for solid tumors. We also computed tumor-specific performance of DrugSense, as shown in Figure 1F. As a negative control, we analyzed PDX transcriptional profiles from solid tumor PDXs with DrugSense trained on liquid tumors, and vice versa. As shown in Supplemental Figure S1J, http://links.lww.com/HEP/I17, the precision was close to the random value, as expected. These data show that DrugSense achieves a better than random performance for 4 out of the 5 pediatric tumor subtypes.

Finally, we compared the performance of DrugSense with other computational methods that have been previously published. We used the benchmarking data from a recent international challenge that focused on adult cancers.^15^ As shown in Supplemental Figure S5, http://links.lww.com/HEP/I17, DrugSense achieved comparable results to the other methods on adult cancers, despite its simplicity and easy interpretability.

### Computational identification of potentially therapeutic drugs for high-risk patients with HB

**FIGURE 2:**
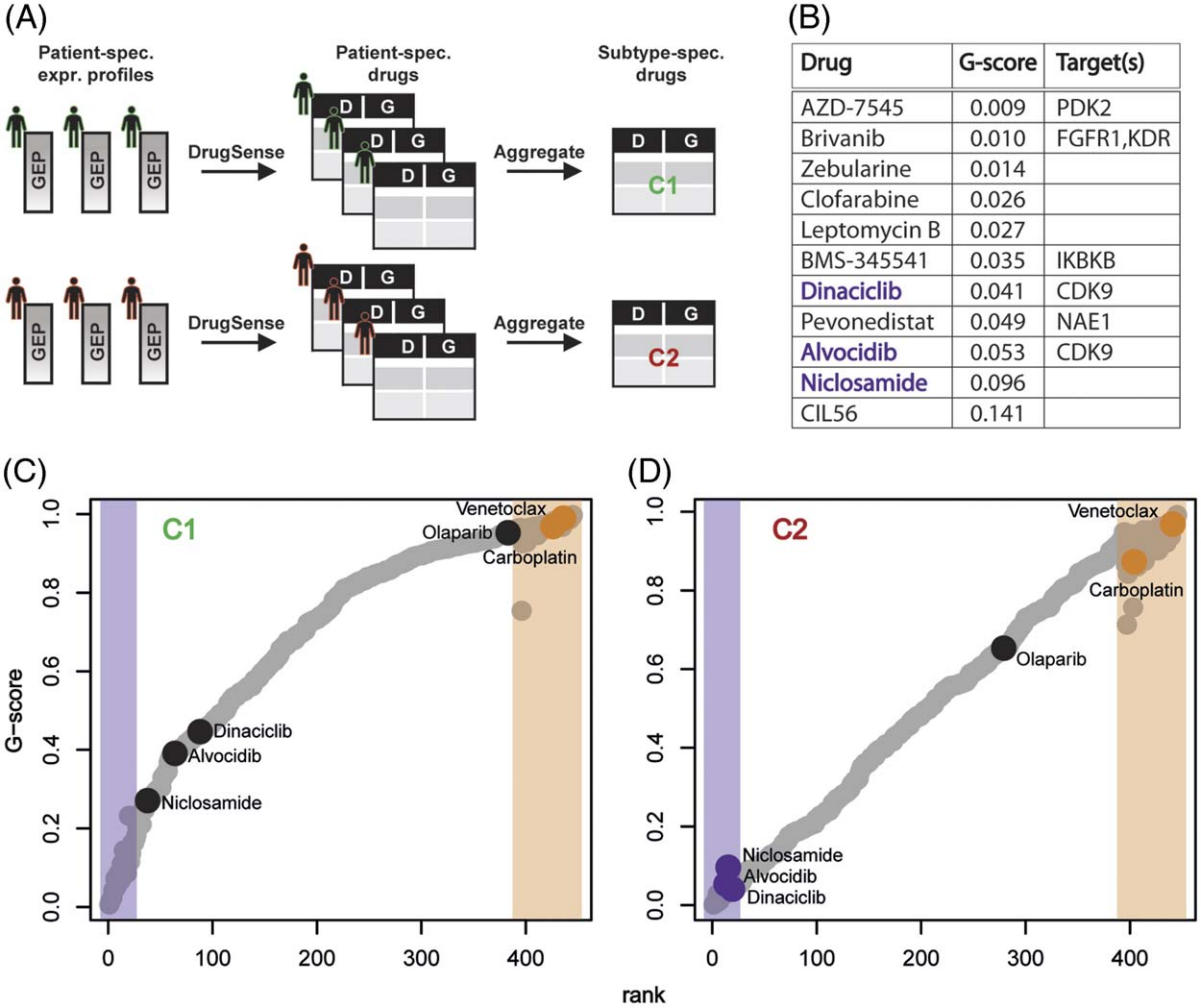
DrugSense identifies drugs specific for the aggressive C2 hepatoblastoma subtype. (A) Transcriptional profiles in a cohort of 24 patients with hepatoblastomawhose tumor belonging either to the C1 subtype (n=18) or the C2 subtype (n=8). Each profile was separately analyzed by DrugSense yielding a ranked list of drugs for each patient. The patient-specific ranked lists of drugs for patients of the same subtype (C1 or C2) were aggregated together to yield a single list of drugs. At the end of the process, only 2 subtype-specific ranked lists of drugs remain, one for the C1 subtype and one for the C2 subtype. (B) The 11 drugs predicted to be specific for the C2 subtype ordered by median G-score. (C, D) Median G-score across patients with C1 (C) and patients with C2 (D) for each of the 445 drugs. Highlighted regions show the top 20 ranked drugs (purple) and bottom 50 ranked drugs (orange) determined using the Cross-Entropy Monte Carlo algorithm to aggregate the drug-ranked lists of individual patients (Methods). Abbreviations: D, drug; G, G-score; GEP, gene expression profile.

We applied DrugSense to published transcriptional profiles^5^ measured in a cohort of patients with C1 and C2 subtype HB. As depicted in Figure 2A, we first analyzed the individual transcriptional profile of each patient in the cohort to obtain one list of drugs ranked according to their predicted potency for each patient (ie, from small to large G-scores); we used a Cross-Entropy Monte Carlo algorithm to aggregate the drug lists across patients of the same subtypes to obtain only one ranked list of the top 20 drugs (out of 445 drugs) predicted to be most effective for each of the two subtypes (ie, C1 or C2). Interestingly, 11 drugs were found to be specific for patients with C2 HB, as shown in Figure 2B, while the remaining 9 drugs were found to be in common between the 2 lists (Supplemental Figure S6, http://links.lww.com/HEP/I17). As a negative control, we also aggregated the lists of drugs across patients to yield the ‘worst’ 50 drugs found at the bottom of the lists, for each of the two subtypes (ie, C1 or C2).

**FIGURE 3:**
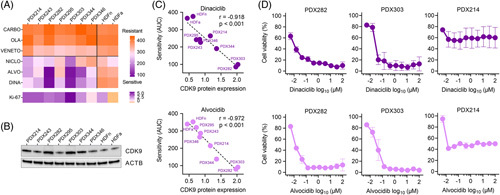
*In vitro* validation of DrugSense in hepatoblastoma cell culture models. (A) A heatmap showing drug sensitivity of the 7 liver cancer models and normal neonatal (HDFn) and adult (HDFa) skin fibroblasts to CARBO, OLA, VENETO, NICLO, ALVO, and DINA. AUC values are the mean out of 2 independent cell viability experiments, each consisting of duplicate measurements. The color scale from orange to purple indicates increasing sensitivity to drug treatment. Ki-67 depicts relative RNA expression of the MKI67 gene normalized to the housekeeping gene TATA-box binding protein. (B) Western blot detection of CDK9 and ACTB in 7 cultures from PDX cell lines established from patients with hepatoblastoma. (C) Correlation of sensitivity (AUC) toward dinaciclib and alvocidib with relative CDK9 protein expression (normalized to ACTB expression) in 7 liver cancer models (PDX) and normal neonatal (HDFn) and adult (HDFa) fibroblasts. Pearson *r* and 2-tailed *p*-values were calculated, and linear regression is given as dashed lines. (D) Dose-response curves of alvocidib and dinaciclib treated cells of the 2 most sensitive tumor models PDX282 and PDX303, as well as the PDX214 model. Error bars stand for standard error of the mean of 2 independent experiments, each consisting of duplicate measurements. Abbreviations: ACTB, beta-actin; ALVO, alvocidib; CARBO, carboplatin; CDK9, cyclin-dependent kinase 9; DINA, dinaciclib; HDF, Human Dermal Fibroblasts; NICLO, niclosamide; OLA, olaparib; PDX, patient-derived xenograft; VENETO, venetoclax.

To experimentally validate the potency of the drugs found by our computational approach, we selected three drugs among the 11 C2-specific top-ranking drugs (ie, the most potent out of 445 drugs) (Figure 2B, D) and 3 drugs predicted to be ineffective in patients with both C1 and C2 (Figure 2C, D) and tested for their potency in 7 PDX cell culture models of HB. These models have been established from a human PDX collection that originates from patients with HB with characteristic clinical and molecular aspects (Supplemental Figure S7, http://links.lww.com/HEP/I17).

Specifically, among the 11 drugs predicted to be most potent in patients with C2, we selected alvocidib and dinaciclib, as these 2 have as a common target the CDK9, which is involved in RNA elongation during transcription,^22^ and niclosamide, an antihelminthic drug that has been recently reported to be effective in blocking proliferation of cancer cells.^23,24^ Among the 2 lists of bottom 50 drugs (ie, 1 for patients with C1 and the other for patients with C2) we found 23 drugs in common between the 2 (Supplemental Table S1, http://links.lww.com/HEP/I17); we thus selected the only 2 drugs (out of 23) that are Food and Drug Administration (FDA)-approved: the widely used chemotherapeutic agent carboplatin, and venetoclax, a drug targeting the antiapoptotic B-cell lymphoma-2 protein. As a third drug to test, we selected the FDA-approved drug olaparib, as this is a PARP1 inhibitor known to inhibit growth in combination therapies in HCC models,^25^ and it is found in the bottom half of the ranked lists for both C1 and C2 patients, and thus predicted not to be effective (Figure 2C, D).

As shown in Figure 3A, the 3 top-ranked drugs: niclosamide, alvocidib, and dinaciclib, all have a strong inhibitory effect on the viability of tumor cells, differently from the bottom-ranked drugs carboplatin, venetoclax, and olaparib. Of note, similar drug concentrations of all drugs had little inhibitory effect on the growth of normal neonatal and adult skin fibroblasts (Figure 3A). These data strongly suggest that DrugSense can predict effective drugs from transcriptomic data with high accuracy for patients with HB.

### Preclinical validation supports CDK9 inhibition as a promising therapeutic approach for HB

**FIGURE 4:**
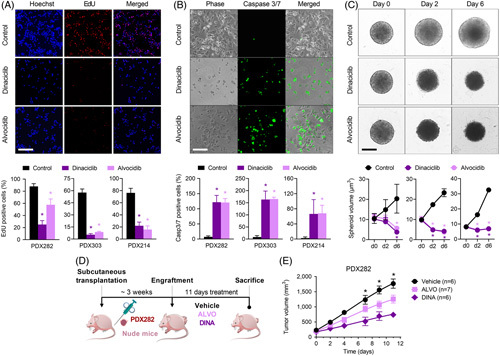
Preclinical in vivo validation of ALVO and DINA in a hepatoblastoma mouse model. (A) Proliferating cells were detected by EdU-staining (red) and quantified in relation to Hoechst 33342-stained nuclei (blue). (B) Apoptotic cells were detected by staining caspase 3/7 activation (green) and quantified in relation to adherent cells (phase contrast). (C) Three-dimensional growth was monitored on the indicated days (d) by calculating spheroid volume. For all experiments in D–F, PDX282 and PDX303, and PDX214 cells were treated with vehicle (CTRL), ALVO, and DINA. Scale bars represent 150 μm, error bars stand for SE of the mean of 2 independent experiments, each consisting of duplicate measurements. *p*-values were calculated using a 2-tailed unpaired Student *t* test. (D) Experimental overview of ALVO and DINA testing in vivo. Immune-compromised mice bearing subcutaneously transplanted PDX282 tumors were i.p. injected with 5 mg/kg body weight ALVO, 20 mg/kg body weight DINA, or vehicle 3 times per week. Mice were killed at day 11 due to maximal tumor size of the control group. (E) Tumor growth in mice treated with either ALVO, DINA, or vehicle. Values correspond to tumor volume and represent means + SEM. Abbreviations: ALVO, alvocidib; CTRL, control; DINA, dinaciclib; EdU, ethynyl deoxyuridine; PDX, patient-derived xenograft.

DrugSense revealed the 2 nonspecific CDK9 inhibitors alvocidib and dinaciclib as the most promising drugs to be tested preclinically. The expression of the target CDK9 was corroborated at the protein level in the 7 PDX models (Figure 3B), being highest in cells derived from the HB models PDX303 and PDX282 that showed the highest sensitivity to alvocidib and dinaciclib (Figure 3C). A subsequent 10-point dose-response curve revealed a dramatic drop in cell viability already at concentrations in the nanomolar range (Figure 3D). To rule out that these 2 models are intrinsically more sensitive to these drugs because of a possible interference with the cell cycle, we also tested PDX214, which has a similar Ki-67 proliferation score as PDX282 (Figure 3A), but lower CDK9 protein expression (Figure 3B). As expected, both alvocidib and dinaciclib have less effect in PDX214 cells in terms of cell viability than in the cells of the 2 CDK9 high expressing PDX282 and PD303 models, thereby underscoring the on-target effect of alvocidib and dinaciclib on CDK9. At the cellular level, reduced viability was associated with a significant decrease in proliferation (Figure 4A) and a simultaneous induction of apoptosis (Figure 4B). Moreover, the three-dimensional growth of the tumor models as spheroids was also dramatically impaired by treatment with alvocidib and dinaciclib (Figure 4C).

Encouraged by the promising results obtained in HB cells derived from PDX models, we tested the efficacy of both drugs *in vivo* by using the prototypical HB282 PDX model after subcutaneous transplantation into nude mice (Figure 4D). This model originates from a primary tumor of a 1-year-old patient, without multifocal and metastatic disease, harbors a CTNNB1 mutation as well as the C2 subtype of the 16-gene signature, and shows a medium proliferation score (Figure 3A and Supplemental Figure S7, http://links.lww.com/HEP/I17). i.p. application of alvocidib and dinaciclib on 3 days per week resulted in a significant reduction of the tumor volume, but also in some intolerances in the form of weight loss and unexpected death (Figure 4E and Supplemental Figure S8, http://links.lww.com/HEP/I17). Collectively, these data qualify CDK9 inhibition as a promising therapeutic approach for HB.

### CDK9 protein is overexpressed in patients with aggressive C2 tumors and associated with poor survival

**FIGURE 5:**
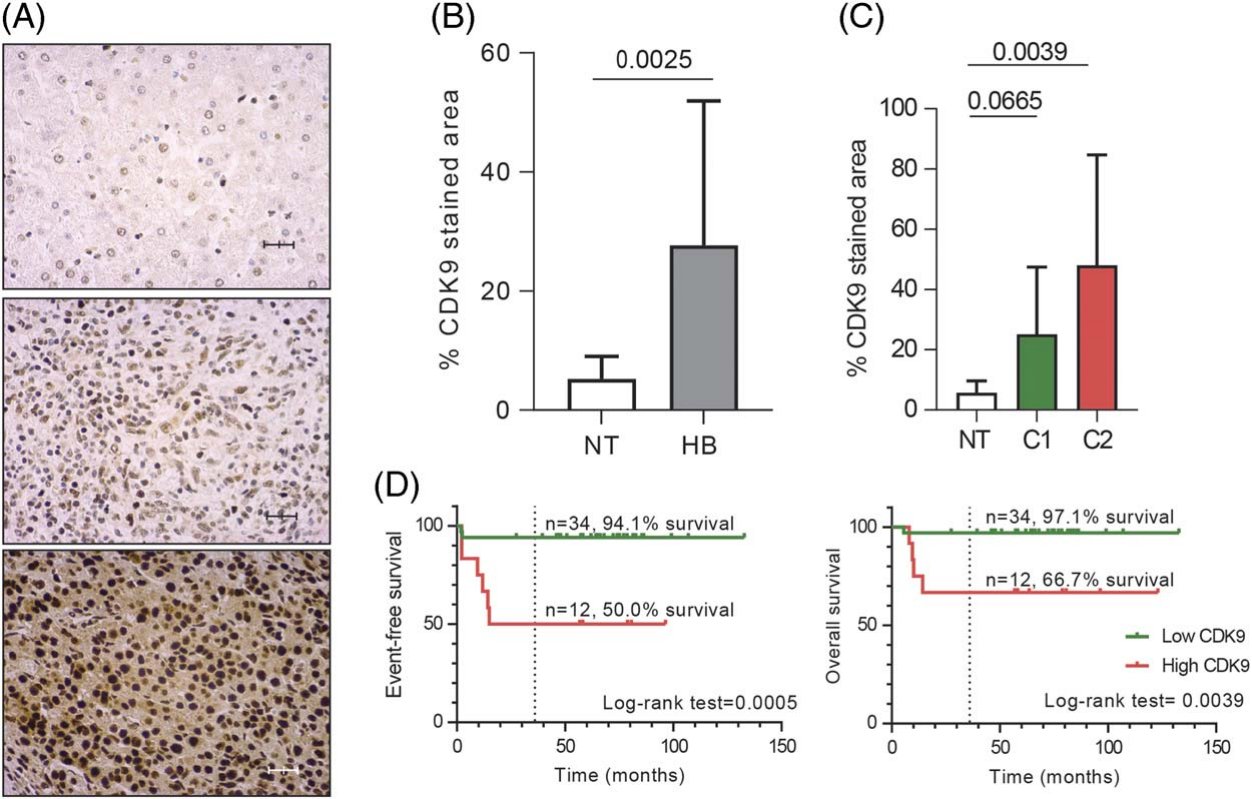
CDK9 is overexpressed in patients with poor prognosis. (A) Representative immunohistochemistry images of CDK9 in NT adjacent liver tissue (top) and HB with low (middle) and high (bottom) staining of CDK9. ×40 magnification. Scale bar: 10 µm. (B) Bar plot of CDK9 staining in tumor (T, n=46) and NT (n=12) samples. *p*-value calculated with Student *t* test. (C) Bar plot of CDK9 staining in NTs and the 2 main transcriptomic HB subgroups (C1, n=29; C2, n=5). *p*-value calculated with ANOVA test. (D) Kaplan-Meier curves of event-free survival (left) and overall survival (right) analysis of patients stratified according to CDK9 tumor staining. The cutoff was set at the percentile 75 of tumor CDK9 staining. Abbreviations: CDK9, cyclin-dependent kinase 9; HB, hepatoblastoma; NT, nontumor.

We measured CDK9 protein expression in the SIOPEL-3 cohort of patients with HB.^7,26^ The main features of the 46 patients with HB are detailed in Supplemental Table S2, http://links.lww.com/HEP/I17. The CDK9 expression levels were determined by immunohistochemistry in 46 tumor and 12 nontumor liver tissues from postchemotherapy surgical specimens. CDK9 staining was weak in nontumor samples, while it was present at heterogeneous intensities across the tumor samples (Figure 5A). Overall, HB showed a significant increase of CDK9 staining as compared with nontumors adjacent livers used as controls (*p*=0.0025; Figure 5B). We then stratified patients’ tumors according to CDK9 staining. Interestingly, CDK9 overexpression, defined as a staining above the 75th percentile, was significantly associated with clinical and pathological parameters of poor prognosis, as reported in Table 1, such as patients’ age above 8 years (*p*=0.003), high-risk stage defined by the Children’s Hepatic tumor International Collaboration risk stratification (*p*=0.009) and an immature main epithelial component (*p*=0.001). In addition, we classified 38 out of 46 patient tumors in the 2 main HB subtypes (C1 and C2) according to the transcriptional expression level of the 16-gene signature^6^ using Nanostring technology. As a result, we classified 31 and 7 tumors belonging to the C1 and C2 molecular subtypes, respectively. The C2 tumors (poor prognosis) but not C1 tumors (good prognosis) presented a significantly higher CDK9 staining than nontumor liver samples by immunohistochemistry (*p*=0.0039, Figure 5C). Finally, we performed a survival analysis to study the impact of CDK9 expression on patients’ outcomes. Kaplan-Meier curves in Figure 5D showed that patients with high CDK9 staining in tumors have a poor outcome when compared to patients with low CDK9 staining, with 3-year event-free survival probabilities of 94% and 50%, respectively (Log-rank=0.0005, Figure 5D). The same finding is confirmed when considering overall survival; patients stratified according to low and high CDK9 tumor staining have a 3-year overall survival probability of 97% and 66.7%, respectively (Log-rank=0 0.0039). In addition, elevated levels of CDK9 staining were associated with older patient age (*p*=0.003), a more immature main epithelial component (*p*=0.01) and advanced clinical risk stratification (*p*=0.009), see Table 1 for details. Therefore, CDK9 could be an optimal therapeutic target for aggressive C2 tumors.

**TABLE 1:** Statistical association between CDK9 staining and clinical features of patients with HB

	Cdk9 staining		
	Low	High	*p*
Clinical features			
Age>8 y	—	—	—
Yes	0	4	0.003
No	34	8	—
Sex			
Female	16	8	0.321
Male	18	4	—
Multifocality			
Yes	27	13	0.129
No	1	3	—
Clinical classification CHIC-HS			
VL-L	1	0	0.009
I	29	6	—
H	3	6	—
AFP plasma levels			
N Mean + SD	34 595,248 ± 965	12 317,672 ± 315	0.149^a^
Metastasis			
Yes	3	4	0.069
No	30	8	—
HB pathological features			
Histology			
Epithelial	15	4	0.735
Mixed	19	8	—
Main epithelial component			
Nonfetal	2	6	0.001
Fetal	32	5	—

^a^ *p*-values were calculated using *t* test.

*Note:* CDK9 expression cutoff was set at 40.55 (Third quartile tumor values). *p*-values were calculated using Fisher exact test.

The nonfetal main epithelial component includes crowded fetal, macrotrabecular, and embryonal histological subtypes.

Abbreviations: AFP, Alpha-Fetoprotein; CDK9, cyclin-dependent kinase 9; CHIC-HS, Children’s Hepatic tumors International Collaboration; H, high; HB, hepatoblastoma; I, intermediate; L, low; VL, very low.

## DISCUSSION

We developed DrugSense, a companion online tool (https://drugsense.tigem.it), which leverages large publicly available collections of GEPs to predict drug sensitivity from a tumor transcriptional profile. More sophisticated approaches have been recently presented in the literature to predict drug response leveraging genomics and transcriptomics profiles, which differ from ours both in methodology and data types used in the training.^27^ For example, the Ideker lab^28^ introduced a machine learning approach that involves pretraining of a general predictive model of drug in CCLs, but then uses the “few-shot” learning to make predictions for a specific type of human tumors. Few-shot learning requires a set of well-characterized clinical samples exposed to the drugs, which hampers its application for rare tumors such as HB. The Califano lab^29^ developed a sophisticated transcription-based oncology platform for predicting in vivo response to antineoplastics based on an ensemble of advanced computational approaches rooted in Information Theory. The need of transcriptional profiles of high-quality tumor-matched cohorts required by this platform makes its application to rare pediatric cancers challenging.

We applied DrugSense to find therapeutic drugs effective against the aggressive C2 subtype of HB. Two CDK9 inhibitors, namely alvocidib and dinaciclib, were thus identified and extensively validated *in vitro* and *in vivo* in PDX mouse models; moreover, CDK9 expression levels were found to be predictive of clinical outcome in a cohort of patients with HB.

An increasing number of CDK9-inhibiting small molecules have recently been introduced. First-generation and second-generation CDK9 inhibitors, such as alvocidib and dinaciclib, have shown promising antitumor activity. We could also prove high CDK9 protein expression levels in PDX cells. Moreover, we found pronounced CDK9 expression in tumor samples of patients with HB, with high expression being associated with clinical risk factors such as increased patients’ age and immature histology, but most importantly, poor prognosis.

In the current study, we show that CDK9 inhibition by dinaciclib and alvocidib impeded short-term and long-term proliferation and induced apoptosis in high-risk pediatric liver cancer models. Most importantly, our data demonstrated superior inhibition of cell proliferation by low nanomolar concentrations of alvocidib or dinaciclib as compared to carboplatin, which is given to patients with HB nowadays.^30^ However, at least in our experimental setting, although using comparable concentrations as in earlier studies described to be safe,^31^ both drugs caused side effects such as weight loss and unexpected death (Supplemental Figure S8, http://links.lww.com/HEP/I17). Nevertheless, dinaciclib has been already safely applied in dose-escalation studies in patients with advanced malignancies^32^ and its use is currently evaluated in many clinical trials for various human cancers. However, we cannot rule out that the observed effect in our PDX model is attributable to the pan-inhibitory effect of both drugs on other CDKs, which might be more relevant for HB.

Interestingly, we also found a significant enrichment for MYC target genes in gene expression biomarkers of sensitivity found by DrugSense for alvocidib and dinaciclib (Supplemental Figure S9, http://links.lww.com/HEP/I17). As it has been described that HB highly depends on MYC,^5^ and that proliferation of hepatocellular cancer cells that also express high levels of MYC could be abrogated by CDK9 gene silencing,^33^ it could be speculated that interference of the CDK9/MYC relationship underlies the molecular mechanism behind the effectiveness of both drugs in HB. However, a recent study suggested the dispensability of CDK9 in YAP-driven HB cells, even though authors clearly detected a significant reduction of proliferation of HepG2 cells on CDK9 silencing.^34^ Nevertheless, our data on viability, proliferation, and apoptosis together with studies on HCC^33^ and other YAP-driven cells^35^ strongly suggest that HB cell survival requires CDK9.

## METHODS

For further details, please refer to the Extended Material and Methods in the Supplemental Material, http://links.lww.com/HEP/I17.

### Training phase of DrugSense: building the DGCPs

We downloaded the basal GEPs [bulk RNA-sequencing data (v21Q4)] of 1375 CCLs from the Cancer Cell Line Encyclopaedia^9^ and drug response data across the CCLs from the CTRPv2. To identify genes whose expression was correlated with drug potency, for each gene and for each drug, we computed the PCC between the expression of the gene and the effect of the drug expressed in terms of AUC across the CCLs. For each drug, we generated a DGCP by ranking genes according to their PCC in descending order. In total, we obtained 445 DGCPs, corresponding to 445 drugs, for solid tumors and 414 DGCPs, corresponding to 414 drugs, for liquid tumors.

### Application of DrugSense: computation of the G-score

DRUGSense applies 4 distinct GSEA^19^ for each drug to be tested, as schematized in Figure 1B. To compute the *p*-value associated with each ES for each of the 4 GSEAs, DrugSense generates a null distribution of ESs by applying GSEA to 10,000 randomized profiles by shuffling 25% of the gene labels of the tumor GEP. This set of four *p*-values, 1 for each GSEA, is averaged using the geometric mean to assess the overall sensitivity of the drug (ie, the G-score). As the 4 GSEA analyses may be potentially correlated, we also applied Brown method for *p*-value integration, which specifically accounts for correlations, as an alternative approach. Despite differences in the absolute values of the resulting *p*-values, we observed that the PPVs were identical for both methods, as shown in Supplemental Figure S3A, http://links.lww.com/HEP/I17. This happens as the ranked list obtained by sorting according to Brown *p*-values is nearly identical to those obtained when sorting according to the geometric mean of *p*-values.

### Validation of DrugSense on CCLs

Precision of DrugSense in predicting drug sensitivity was evaluated in CCLs using 2 publicly available data sets of bulk GEPs of untreated cells. To determine if a cell line was sensitive or not to a specific drug, we exploited the CTRPv2 drug response dataset, which includes drug response in terms of AUC of the dose-response of CCLs. Drug potency across solid and liquid CCLs was kept apart and analyzed separately. The PPVs (PPV= True Positive/ [True Positive + False Positive] were computed by binning cell line/drug pairs in percentiles. The PPVs were normalized against the PPV obtained from a random ordering of cell line/drug pairs.

### Validation of DrugSense on PDX data

Precision of DrugSense in predicting drug sensitivity was evaluated in PDX mouse models following the same pipeline as described above for CCLs, but this time using data from the PCAT^21^ data portal on the drug response data of 39 PDX models treated with one or more drugs for which a DGCP was available in DrugSense.

### Benchmarking of DrugSense in the National Cancer Institute-Dialogue for Reverse Engineering Assessment and Methods challenge

To benchmark DrugSense’s ability to predict drug response against other approaches, we used the data provided by the National Cancer Institute-Dialogue for Reverse Engineering Assessment and Methods drug prediction challenge.^15^ We compared DrugSense’s performance with that of the nine other drug sensitivity prediction algorithms present in the National Cancer Institute-Dialogue for Reverse Engineering Assessment and Methods challenge that used the same training data, that is, gene expression (e), RNA-sequencing (n), and outside information.

### Application of DrugSense to HB

Microarray-based gene expression data of 25 HB samples in Cairo et al^5^ were first normalized using the robust multiarray average method (R package affy, v1.72.0)^36^ and aligned with the cell line expression profiles of the Genomics of Drug Sensitivity in Cancer data set. We then applied DrugSense to these processed profiles comprising 25 HB samples to predict the potency of each drug in each sample. Drugs were ranked by G-score in ascending order according to their predicted potency, and individual rankings related to either the C1 or the C2 samples were aggregated using the *RankAggreg* function.^37^

### Gene ontology enrichment analysis of alvocidib and dinaciclib biomarkers

The 177 biomarkers of sensitivity in common between alvocidib and dinaciclib were analyzed by means of Gene Ontology Enrichment Analysis using as gene sets either the C5 Gene Sets, or the Hallmark Gene Sets downloaded from the MSigDB3.0. Gene Ontology Enrichment Analysis was performed with the *clusterProfiler* package^38^ in R statistical environment. The threshold applied for statistical significance was False Discovery Rate <0.05.

### Cell culture

Seven PDX cell lines (PDX214, PDX243, PDX282,^39^ PDX295, PDX303, PDX344, and PDX346) were kindly donated by Stefano Cairo (XenTech, Evry, France). Two dermal fibroblast cell lines adult HDFa and neonatal HDFn (The American Type Culture Collection) were also included as noncancerous controls.

### Viability assay

MTT [3-(4, 5-dimethylthiazol-2-yl)-2, 5-diphenyltetrazolium bromide] (Sigma-Aldrich, St. Louis, MO) viability assay was performed to determine drug responses of the cells. A total of 5×10^4^ cells/well were seeded in a 96-well plate 24 hours prior to drug exposure.

### Apoptosis assay

Apoptotic cell count was determined by means of CellEvent Caspase-3/7 Green Detection Reagent (Thermo Fisher) according to the manufacturer’s instructions. A total of 2×10^5^ cells/well were seeded in a 24-well plate 24 hours prior to drug exposure and measured following 24 hours dinaciclib (0.1 µM) or alvocidib (0.1 µM) exposure.

### Proliferation assay

Click-iT EdU Cell Proliferation Kit (Thermo Fisher) was applied according to the manufacturer’s instructions for the detection of proliferating cell portions. A total of 2×10^5^ cells/well seeded in a 24-well plate. Next day, the cells were labeled with 100 µM ethynyl deoxyuridine and exposed to dinaciclib (0.1 µM) or alvocidib (0.1 µM) for 24 hours.

### Western blot analysis

Whole cell lysates were extracted from 3 × 10^7^ cells grown in 100 mm cell culture petri dishes for 24 hours. Cell pellets were incubated in cell lysis buffer. 20 µg whole cell lysates were separated by means of SDS-gel electrophoresis, using Novex WedgeWell 8% pre-cast tris-glycine gels (Thermo Fisher). The membrane was incubated overnight at 4 °C in anti-CDK9 antibody (Sigma-Aldrich, #HPA006738) diluted 1:1000. Anti-beta-actin in a 1:10,000 dilution (Cell Signaling Technologies, Danver, MA, #4967S) served as a loading control. Following the incubation with a secondary goat-anti-rabbit HRP antibody (Dako Denmark, Glostrup, Denmark, # P0448) in a 1:5000 dilution, proteins were detected by the ChemiDoc XRS+ imaging system (Bio-Rad).

### Spheroid formation

Cells were seeded at a density of 1000 cells/well into ultra-low attachment round-bottom 96-well plates (Corning, Corning, NY). After 5 days of incubation, established spheroids were exposed to dinaciclib (0.1 µM), alvocidib (0.1 µM), or DMSO control in medium. Spheroid images were captured at the indicated timepoints with the EVOS M7000 imaging system (Thermo Fisher). Spheroid volumes (V) were calculated by [V (µm^3^)=(length (µm) × width (µm)^2^)/2] formula.^40^

### In vivo study

Animal studies were carried out by XenTech according to studies.^39,41^ PDX282 tumors were implanted into Athymic nude-Foxn1nu mice. Mice with subcutaneously growing tumors between 75 and 288 mm^3^ were allocated to each treatment arm as 7 mice/group. Mice were treated with vehicle, alvocidib (5 mg/kg), or dinaciclib (20 mg/kg) by i.p. injection for 5 subsequent days per week. Tumor volume was evaluated by measuring tumor diameters with a caliper, 2 or 3 times a week during latency and treatment period.

### Patients and samples

The study included a total of 58 tumor and nontumor samples from 46 patients with HB (Supplemental Table S2, http://links.lww.com/HEP/I17). All samples were collected in accordance with European and Spanish law and institutional ethical guidelines. Informed consent was obtained in accordance with European Union guidelines for biomedical research. The study conformed to the ethical guidelines of the 1975 Declaration of Helsinki. HB diagnosis was defined by expert pathologists. Patients included in the study were enrolled in the SIOPEL-3 clinical trial. Human Ethics Committee of the Hospital Universitari Germans Trias i Pujol, references: PI17-079 and PI-18-203.

### Immunohistochemistry

Four-micrometer-thick tissue sections from paraffin blocks were baked for 20 minutes at 65 °C. Sections were then incubated with primary antibody, CDK9 (Sigma-Aldrich catalog number HPA006738), 1/300, overnight, at 4^ ^°C, in a humid chamber. Later, rabbit linker (Agilent) and HRP polymer conjugated secondary antibody (Visualization reagent, Agilent) were applied for 15 minutes and 1 hour, respectively, at room temperature in a humid chamber. Percentage of tumor-stained area was calculated by examination of 3–6 random high-power fields (×40) and quantified with specific thresholds using ImageJ software v.45.s (National Institutes of Health).

### NanoString nCounter

To classify tumors according to the C1/C2 classification, 38 out of our 46 HBs (for 8 tumors, no additional tissue was available) were profiled using the NanoString nCounter Technology with a manually curated list of 18 genes, including HB markers and genes of key signaling pathways (Supplemental Table S3, http://links.lww.com/HEP/I17).

### Statistical analysis

To study the association between CDK9 immunostaining with clinical and pathological features, Fisher tests and Student *t* tests were used according to convenience. The Kaplan-Meier method was used to compare the impact on patient event-free survival or overall survival of the differential tumor expression of CDK9, calculating survival curves and log-rank tests. Statistical analysis was performed with GraphPad Prism 7 for Windows (La Jolla, CA) and the IBM SPSS statistics for Windows, version 15 (Chicago, IL).

## Supplementary Material
