# Supplementary material for: Computational drug prediction in hepatoblastoma by integrating pan-cancer transcriptomics with pharmacological response
Source: Hepatology. 2023 Sep 20;80(1):55–68. doi: 10.1097/HEP.0000000000000601 (PMC11185924; doi:10.1097/HEP.0000000000000601)
Supplement: Supplementary file 1 [file hep-80-055-s001.docx]

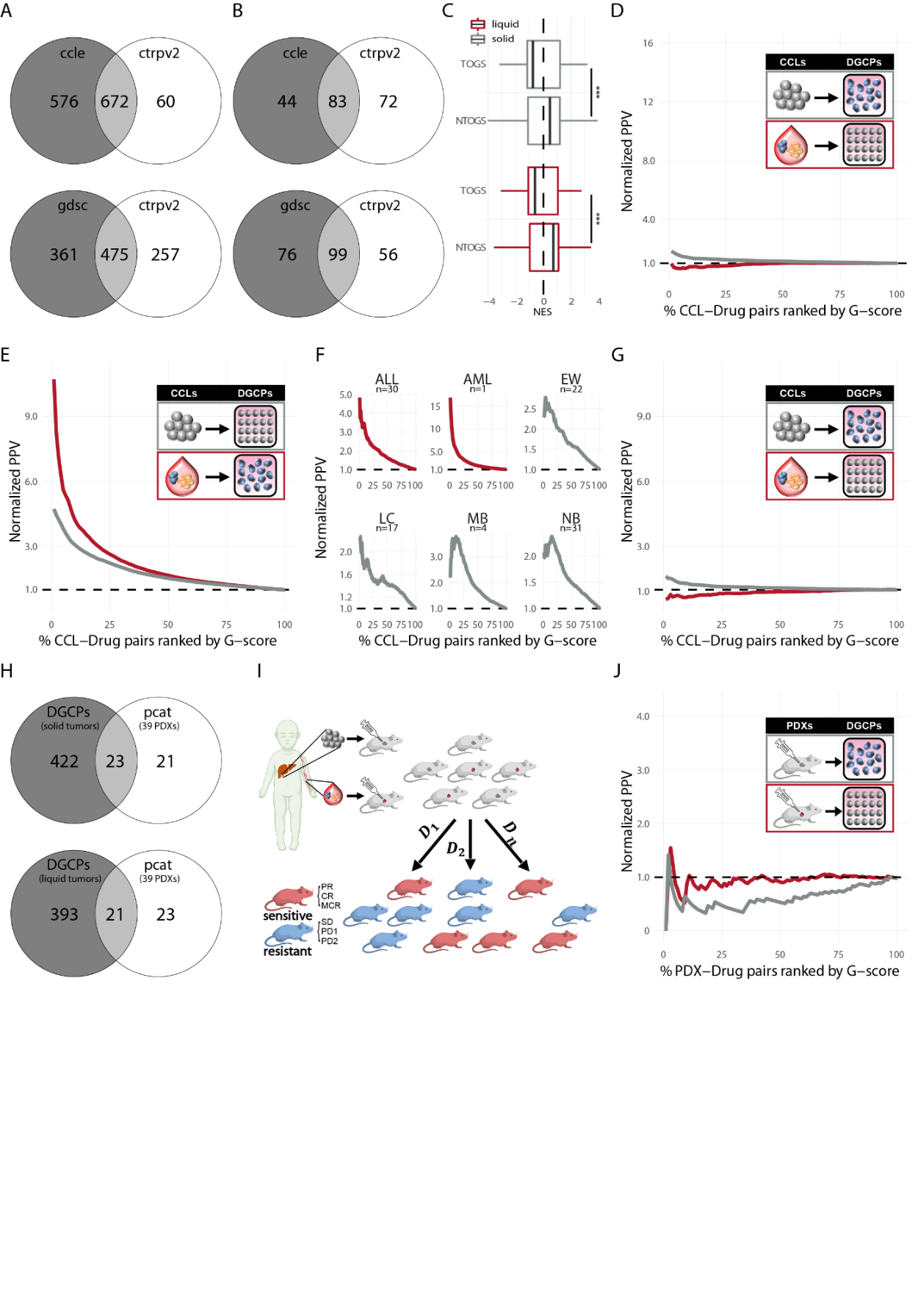


**Supplementary Figure S1** **DrugSense Benchmarks.** **(A,B)** Two-set Venn diagrams showing the number cancer cell lines (CCLs) shared between CCLE gene expression dataset (or GDSC dataset) and the CTRPv2 drug response dataset, for solid **(A)** and liquid **(B)** cancer cell lines. **(C)** Boxplots representing the distribution of Normalised Enrichment Scores (NES) computed by GSEA on the DGCPs when using as input either genes belonging to the same pathways as the drug target (Target Ontology Gene Sets - TOGS), or genes belonging to other pathways (Non-Target Ontology Gene Sets - NTOGS). Results refer to solid or liquid CCLs. **(D)** Positive Predictive Values (PPVs) obtained by DrugSense when using the “wrong” DGCPs (i.e., when swapping solid with liquid) with the CCLE gene expression dataset against the CTRPv2 drug potency gold standard. **(E)** PPVs obtained by applying DrugSense to the GDSC transcriptional data against the CTRPv2 drug potency gold standard. **(F)** PPVs for CCLs from six distinct pediatric tumors obtained from the GDSC transcriptional dataset against the CTRPv2 drug potency gold standard. **(G)** PPVs obtained from the GDSC transcriptional dataset against the CTRPv2 drug potency gold standard when swapping solid with liquid DGCPs. **(H)** Two-set Venn diagrams showing the number of drugs shared between the solid or liquid DGCPs and drugs available in the Childhood Cancer Therapeutics Portal (PCAT) dataset. **(I)** Classification of PDX models in sensitive and resistant models upon drug treatment. **(J)** PPVs obtained from the PPTC transcriptional dataset against the PCAT drug response gold standard when swapping liquid and solid DGCPs.


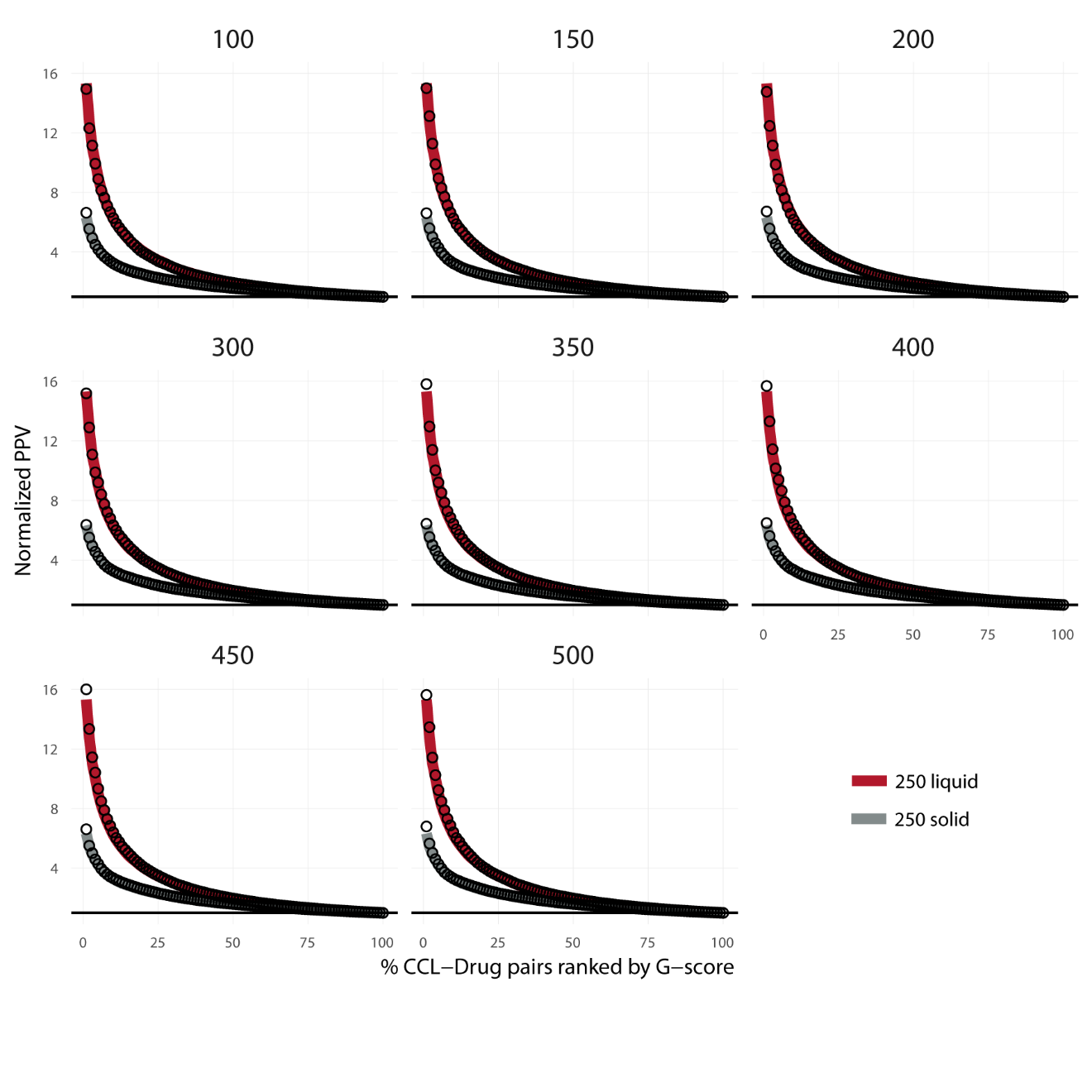


***Supplementary Figure S2: Stability analysis of GSEA.*** DrugSense was applied to the gene expression profiles of cancer cell lines (CCL) present in the CTRPv2 database and the G-score of each drug was computed by systematically varying the number of genes included in the GSEA, ranging from 100 to 500. CCL/drug pairs are ranked according to their G-score in ascending order and reported as percentiles on the x-axis. the Positive Predictive Value (PPV) is normalised against the random PPV (black dashed line) obtained by ordering drug randomly rather than according to their G-score. In each panel, the normalized PPV for the indicated gene set size is reported as empty circles for 672 CCLs from solid and 83 from liquid tumours; in each panel the PPVs for the reference size of 250 genes is also shown as a solid red line (for liquid CCLs) and a solid grey line (for solid CCLs) to make comparison easier.


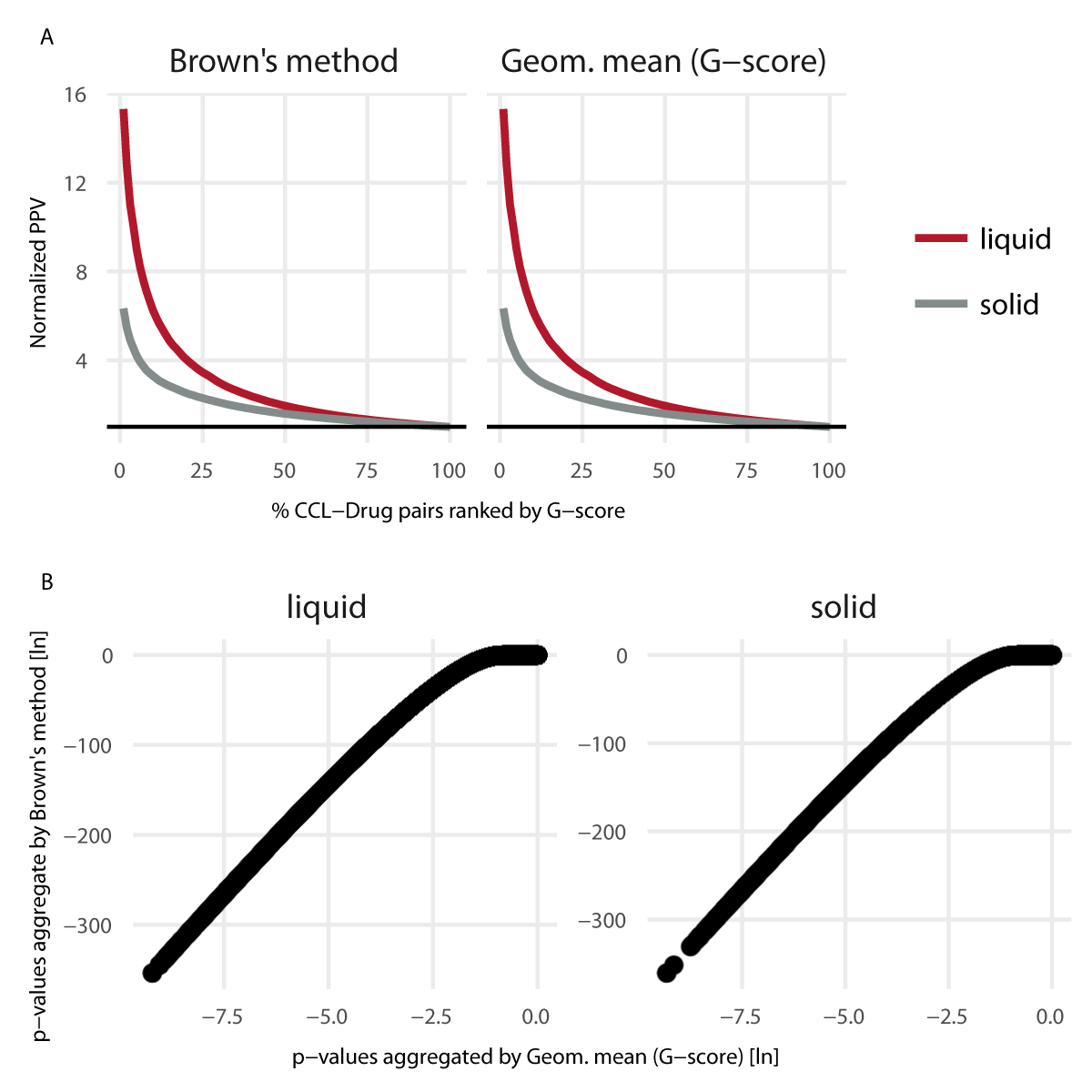


***Supplementary Figure S3 Integration of GSEA p-values using the Brown’s method.* (A)** DrugSense was applied to the gene expression profiles of cancer cell lines (CCL) present in the CTRPv2 database and the resulting p-values for each drug were either aggregated using the geometric mean (G-score) or using the Brown’s method. CCL/drug pairs are ranked according to the Browm’s method (left) or G-score (right) in ascending order and reported as percentiles on the x-axis. The Positive Predictive Value (PPV) is normalised against the random PPV (black dashed line) obtained by ordering drug randomly, and shown as a solid red line for liquid CCLs and a solid grey line for solid CCLs. **(B)** Scatter plot of p-values aggregated by geometric mean (x-axis) vs p-values aggregated by Brown’s method (y-axis) in logarithmic scale for liquid (left) and solid (right) tumours.


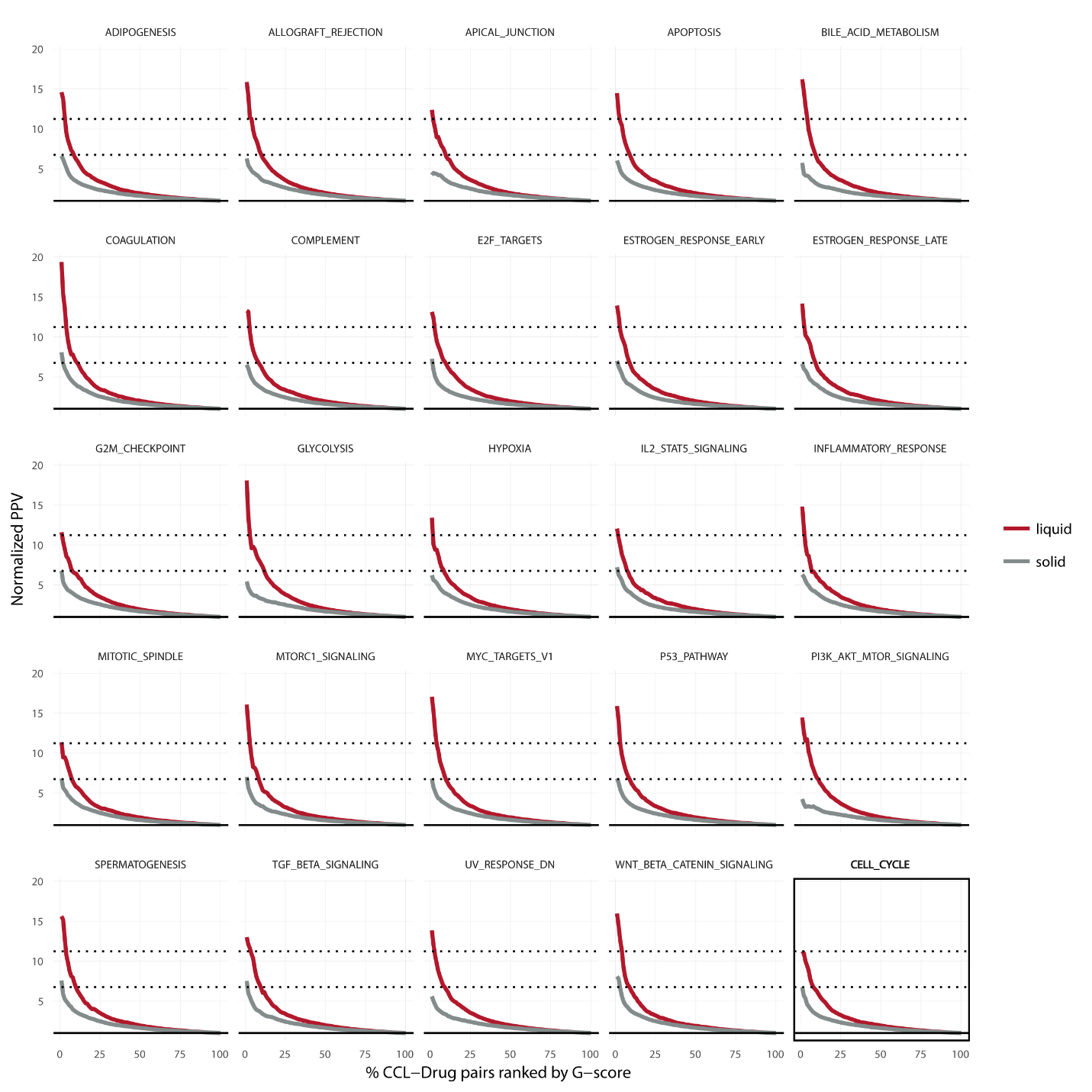


***Supplementary Figure S4: Prediction power of DrugSense for drugs targeting specific pathways.*** Drugs were sorted into classes according to their known targets so that drugs whose targets belong to the same biological pathways fall in the same group. Biological pathways were obtained by manually curated Molecular Signature Database (MSigDB). DrugSense was applied to the gene expression profiles of cancer cell lines (CCL) present in the CTRPv2 database. For each drug class, the CCL/drug pairs are ranked according to their G-score in ascending order and reported as percentiles on the x-axis. The Positive Predictive Value (PPV) for the indicated drug classes is normalised against the random PPV (black dashed line) obtained by ordering drug randomly rather than according to their G-score. Dotted lines correspond to maximum PPVs obtained for the class of drugs targeting the cell cycle.


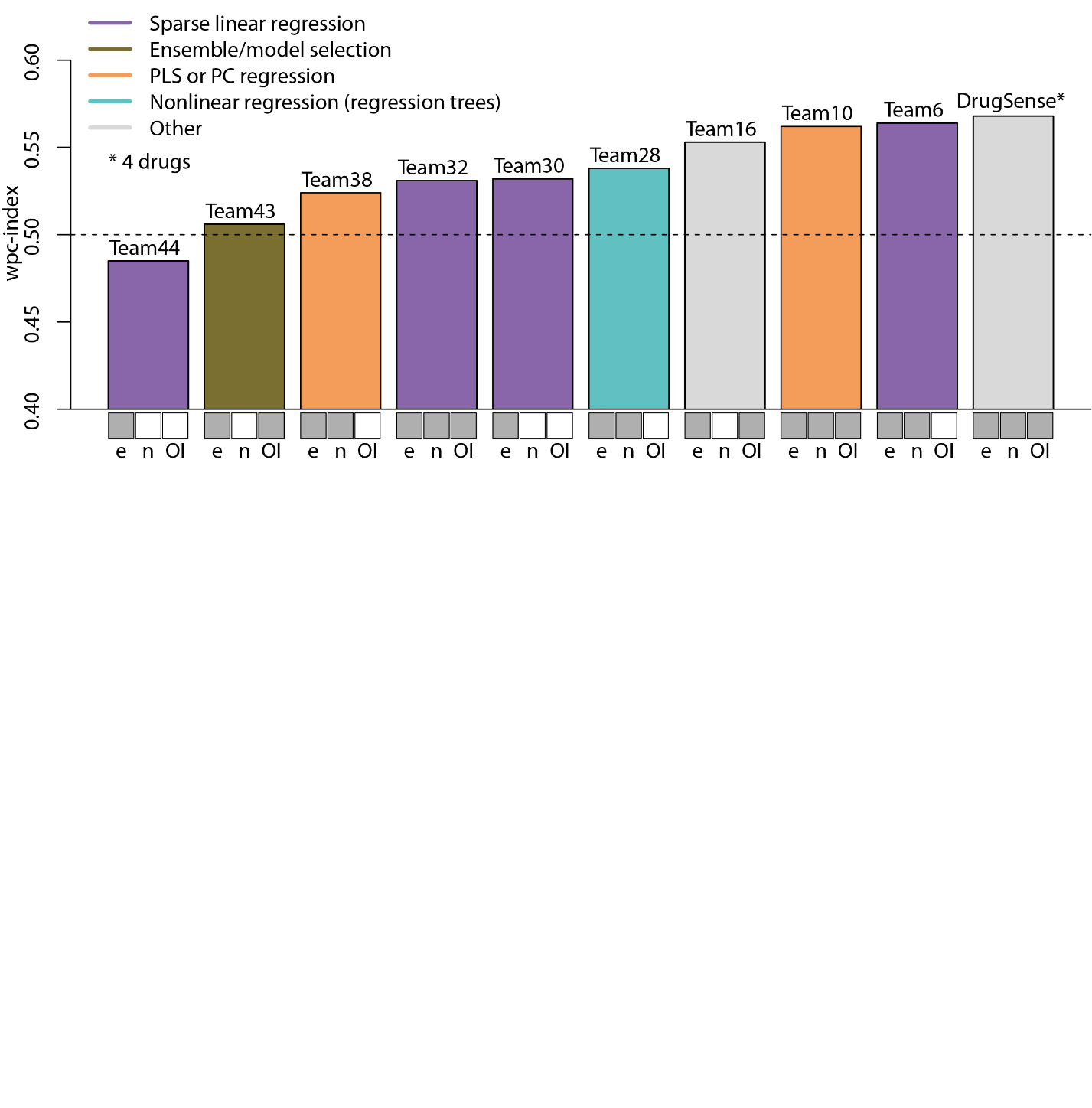


**Supplementary Figure S5 Performance comparison with other drug sensitivity prediction algorithms.** For each team, the underlying methodology and the dataset used are indicated. Team performance is reported as weighted, probabilistic concordance index (wpc-index). The dashed black line represents the mean random prediction score.


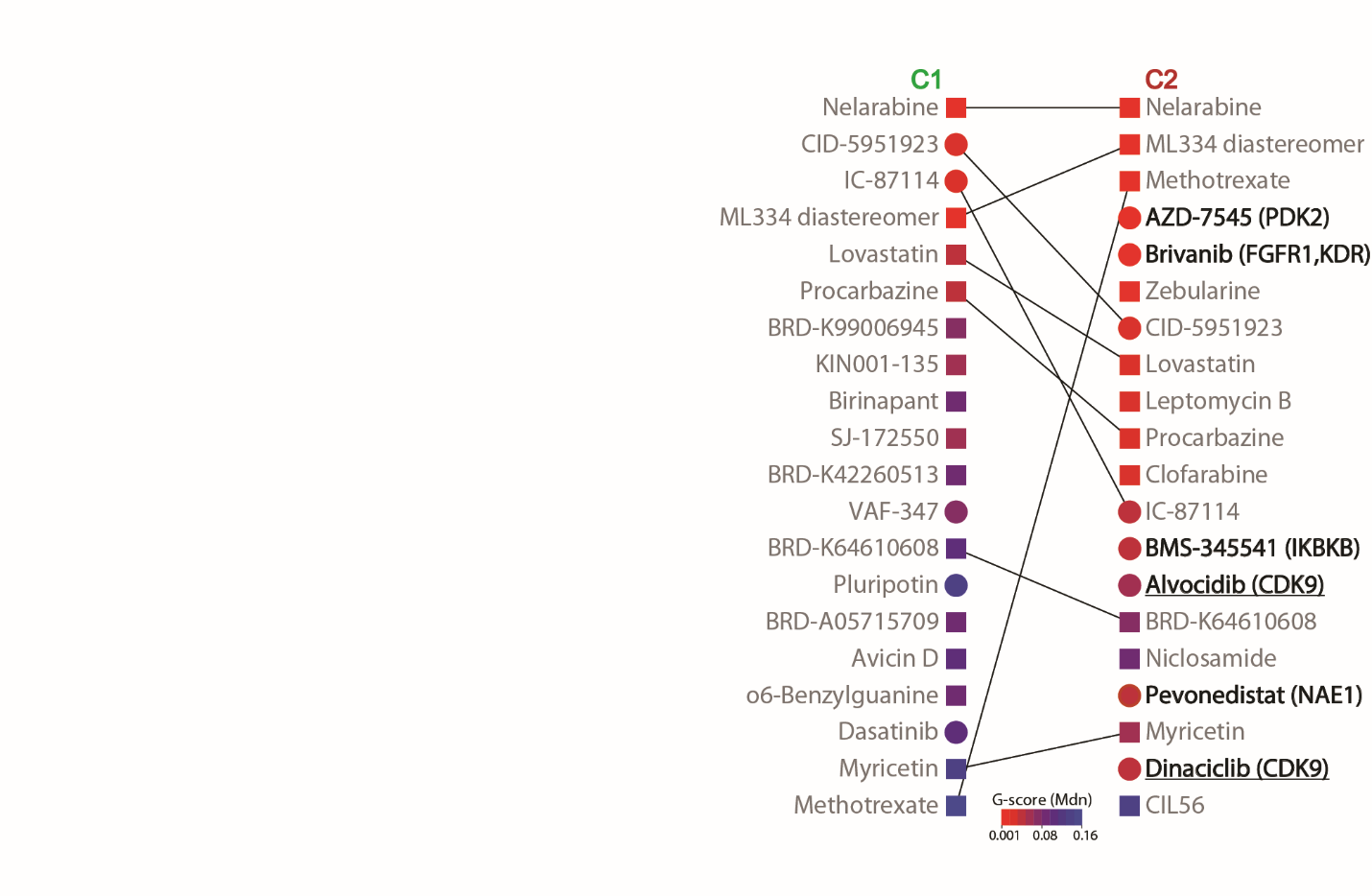


**Supplementary Figure S6** **The first 20 drugs predicted by DrugSense for the C1 and C2 subtypes.** Drugs predicted as effective in both C1 and C2 patients are connected across the two lists, while squares and circles represent non-targeted and targeted drugs, respectively. Targeted drugs predicted uniquely for the C2 subtype are shown in bold with their target(s) reported in brackets. Drugs targeting the same protein are underlined.


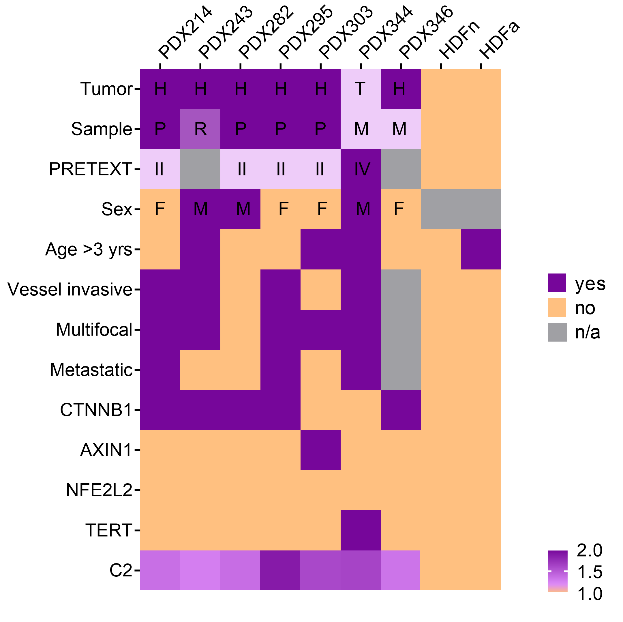


***Supplementary Figure S7 Clinical and molecular characteristics of PDX models.*** Color-coded details on the seven patient-derived xenograft (PDX) tumour models and two fibroblast (HDF) models used for compound validation. Tumor type at diagnosis: H, hepatoblastoma, T, transitional liver cell tumor; model derived from: P, primary tumour, R, recurrence, M, metastasis; II, PRETEXT stage 2, IV, PRETEXT stage 4; F, female, M, male; Age >3 yrs, age of onset of disease > 3 years of age; multifocal, existence of multiple tumour nodules; metastatic, patient presented with metastases at diagnosis; CTNNB1, tumour with mutation in the beta-catenin gene; NFE2L2, tumour with mutation in the nuclear factor erythroid 2-related factor 2 gene; TERT, tumour with mutation in the promoter of the telomerase reverse transcriptase gene; C2, score calculated from the 16-gene signature specific for the C2 subtype.


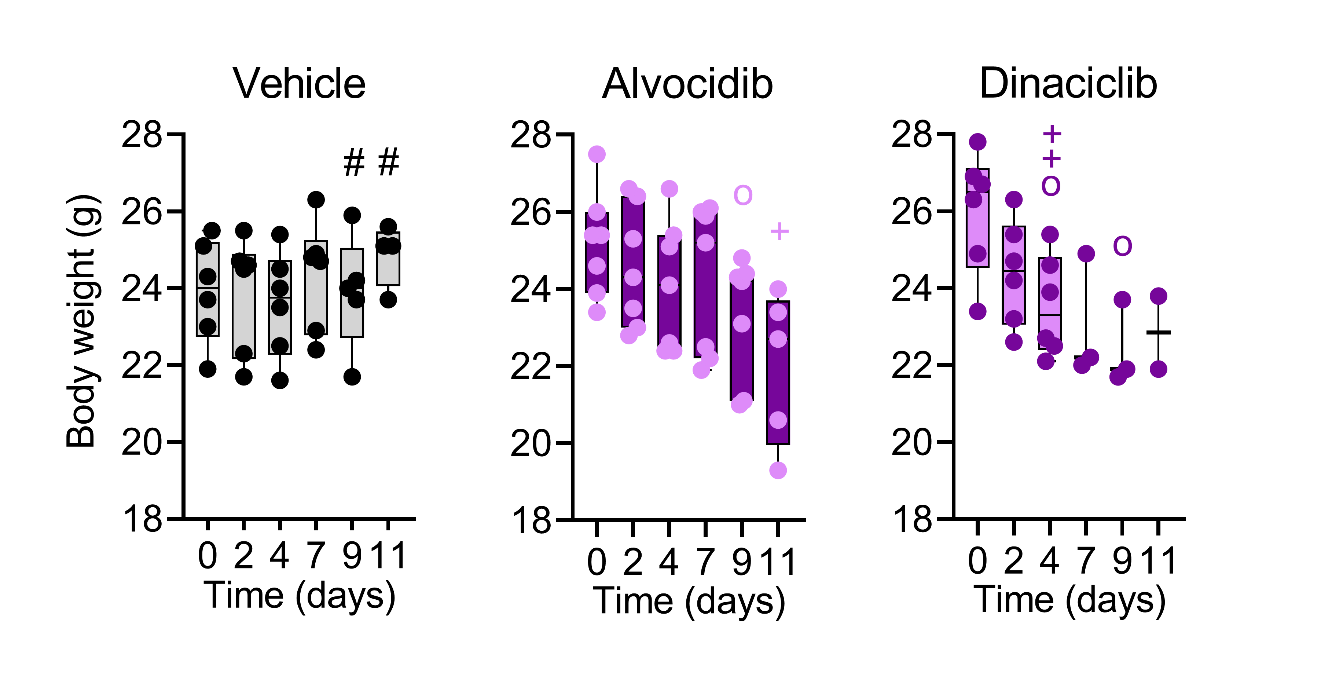


***Supplementary Figure S8*** ***Phenotypic characterisation of PDX mice treated with alvocidib and dinaciclib testing in vivo.*** Immune-compromised mice bearing subcutaneously transplanted PDX282 tumours were intraperitoneally injected with 5 mg/kg body weight alvocidib, 20 mg/kg body weight dinaciclib or vehicle three times per week. Mice were sacrificed at day 11 due to maximal tumour size of the control group. Filled circles correspond to the body weight of individual mice, with the box showing the 25^th^ to 75^th^ percentile, the whiskers the smallest and largest values, and the line in the box the median. o, +, and # denote animals that were excluded from the study at the indicated days of treatment due to body weight loss, unexpected death, and maximal tumour volume, respectively.


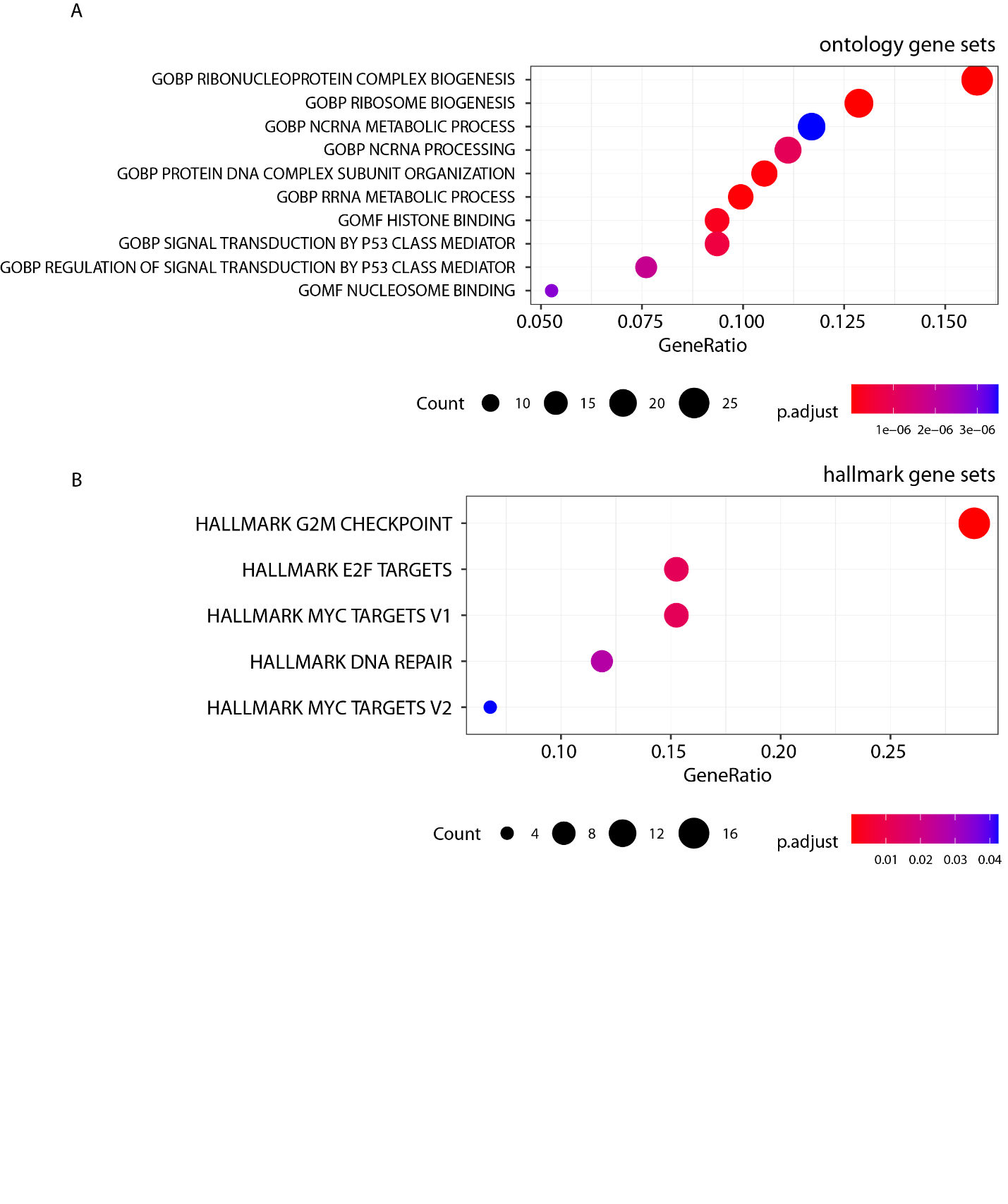


**Supplementary Figure S9 Gene Ontology Enrichment Analysis of the biomarkers of sensitivity of alvocidib and dinaciclib.** Results of the Gene Ontology Enrichment Analysis (GOEA) among the 177 biomarkers of sensitivity to alvocidib and dinaciclib using either the collection of Gene Ontology Gene Sets **(A),** or the Hallmark Gene Sets **(B),** from the MSigDB3.0. Only gene-sets with an FDR < 5% are reported. The 177 biomarkers were found by intersecting the 250 genes at the bottom of the alvocidib’s DGCP with the 250 genes at the bottom of the dinaciclib’s DGCP.

***Supplementary Table 1 The 23 out of the bottom 50 drugs predicted by DrugSense to be in common between the C1 and C2 subtypes.***

|  | **Name** | **FDA** |
| --- | --- | --- |
| **Drugs** | ***Carboplatin*** | ***Y*** |
|  | *Navitoclax* | *N* |
|  | *Serdemetan* | *N* |
|  | *SRT-1720* | *N* |
|  | ***Venetoclax*** | ***Y*** |
|  | *XL765* | *N* |
| **Research Compound** | *Abt-737* |  |
|  | *Avrainvillamide*  *BIX-01294* |  |
|  | *BRD-K26531177* |  |
|  | *BRD-K28456706* |  |
|  | *BRD-K90370028* |  |
|  | *Ch-55* |  |
|  | *Compound 7d-cis* |  |
|  | *Cucurbitacin I* |  |
|  | *JW-74* |  |
|  | *Necrostatin-7* |  |
|  | *NPC-26* |  |
|  | *NSC19630* |  |
|  | *NSC23766* |  |
|  | *Prima-1* |  |
|  | *Repligen 136* |  |
|  | *SZ4TA2* |  |

***Supplementary Table 2 Main clinical and pathological features of the 46 paediatric patients with liver cancer included in the study.***

|  | **HB patients** |  |
| --- | --- | --- |
|  | **N=46** |  |
| Age, months (median, [range]) | 25.9 [0-173] |  |
| Gender (M/F) | 24/22 |  |
| Serum AFP, ng/mL (range) | 522,837 [150-4,700,000] |  |
| Clinical classification CHIC-HS (VL-L/I/H/NA) | 1/35/9/1 |  |
| Tumour stage |  |  |
| PRETEXT stage (I/II/III/IV/NA) | 1/19/23/2/1 |  |
| Multifocality (Y/N/NA) | 4/40/2 |  |
| Vascular Invasion (Y/N) | 6/40 |  |
| Metastasis at diagnosis (Y/N/NA) | 7/38/1 |  |
| HB histology: |  |  |
| Epithelial/Mixed | 19/27/0 |  |
| Main Epith. Comp.: Fetal/Non-Fetal*/NA | 37/8/1 |  |
| Follow-up, months (mean, [range]) | 62.9 [5.5-132.8] |  |
| Outcome: cancer-related deaths (%) | 5 (10.9%) |  |

Abbreviations: AFP, Alpha-fetoprotein; M, Male; F, Female; H/VH, High and Very High risk; L/I, Low and Intermediate risk; N, no; Y, yes; NA, non-available. *Non fetal main epithelial component includes all components different to well differentiated fetal histology (i.e., crowded fetal, embryonal, macrotrabecular, etc)

## Supplementary Table 3 Genes and their corresponding probes included in the NanoString nCounter Technology (n=18).

| AFP | GGAGCGGCTGACATTATTATCGGACACTTATGTATCAGACATGAAATGACTCCAGTAAACCCTGGTGTTGGCCAGTGCTGCACTTCTTCATATGCCAACA |
| --- | --- |
| ALDH2 | CTTCACAAAGGATTTGGACAAGGCCAATTACCTGTCCCAGGCCCTCCAGGCGGGCACTGTGTGGGTCAACTGCTATGATGTGTTTGGAGCCCAGTCACCC |
| APCS | GATCTCTGTCCTCACCAGCCTCCTGGAAGCCTTTGCTCACACAGACCTCAGTGGGAAGGTGTTTGTATTTCCTAGAGAATCTGTTACTGATCATGTAAAC |
| APOC4 | TGGTTCCTCGAATCCAAAGACAGCCTCTTGAAGAAGACCCACAGCCTGTGCCCCAGGCTTGTCTGTGGGGACAAGGACCAGGGTTAAAATGTTCATAAAA |
| AQP9 | TCATCCTCGATGGGAATTCTTGCTAGGTAAGCACTAATAACTCGGCATCTTGACGATAGTCCCATTTGGGTGGTTTCAGCTGCACTATCTGTATGAAATG |
| BUB1 | GAAGATAAAGAAAATGTGGTAGCAAAACAGTGTACCCAGGCGACTTTGGATTCTTGTGAGGAAAACATGGTGGTGCCTTCAAGGGATGGAAAATTCAGTC |
| C1S | CATCCGCTACACTTGTGAGGAGCCATATTACTACATGGAAAATGGAGGAGGTGGGGAGTATCACTGTGCTGGTAACGGGAGCTGGGTGAATGAGGTGCTG |
| CYP2E1 | AGCCGACATCCTCTTCCGCAAGCATTTTGACTACAATGATGAGAAGTTTCTAAGGCTGATGTATTTGTTTAATGAGAACTTCCACCTACTCAGCACTCCC |
| DLG7 | TAAATTATTCTCAGGACTTTCTGTCTCTTCTGAAGGCCCTTCTCAAAGACTTGGAACACCTAAGTCTGTCAACAAAGCTGTATCTCAGAGTAGAAATGAG |
| DUSP9 | CCTGCCTCTTCTGCGACTGTTACTTTTTCTTTGCGGGATGGGGGTGGGGGTTCCCTCTCCAGGTGGTTGTCCAGGCCCATGTCCCGGCCCTGGGTGCTCA |
| E2F5 | AATTGAAGATCTAGAACTGAAGGAAAGAGAACTTGATCAGCAGAAGTTGTGGCTACAGCAAAGCATCAAAAATGTGATGGACGATTCCATTAATAATAGA |
| GHR | TTCATATAGTACAGTCCCCACAGGGCCTCATACTCAATGCGACTGCCTTGCCCTTGCCTGACAAAGAGTTTCTCTCATCATGTGGCTATGTGAGCACAGA |
| HPD | CGATCACCTGGTGAAACACGGTGACGGAGTGAAGGACATTGCGTTCGAGGTGGAAGATTGTGACTACATCGTGCAGAAAGCACGGGAACGGGGCGCCAAA |
| IGSF1 | CACTCACACTGAAAAACGCCCCTTCAAGTGGTCTGAGCCCAGTGAGCCGCTGGAGCTTGTCATAAAAGAAATGTACCCTAAGCCCTTCTTCAAGACATGG |
| NLE | GCGGCAGCAGTGACAGCACACTGAAGGTGTGGGATGTGAAGGCCCAGAAGCTGGCCATGGACCTGCCCGGCCACGCGGATGAGGTATATGCTGTTGACTG |
| RPL10A | GTTTTTGGCCTCAGAGTCTCTGATCAAGCAGATTCCACGAATCCTCGGCCCAGGTTTAAATAAGGCAGGAAAGTTCCCTTCCCTGCTCACACACAACGAA |
| PNN | GTGCGTAATGAAGAACAGAAGGCGGAACAAGAAGAGGGTAAGGTGGCTCAGCGAGAGGAAGAGTTGGAGGAGACAGGTAATCAGCACAATGATGTAGAAA |
| RHOT2 | GGTGCAGGCCAGGCTGCCACTCCGGGAACGCCTTTGCGCCGGGACTTTTTGTTTCTGAAGGCAGTCGATCTGCAGCGGGGCCTTATGCTGCCATGCACTG |

# Extended Material and Methods

**Training phase of DrugSense: building the Drug Gene Correlation Profiles (DGCPs).**

We downloaded the basal gene expression profiles (bulk RNA-sequencing data (v21Q4)) of 1,375 CCLs from the Cancer Cell Line Encyclopaedia (CCLE)^1^. Briefly, the raw counts of each gene were normalized with the *edgeR* package^2^ and transformed into log10(CPM+1). Lowly expressed genes and genes whose entropy was in the 5^th^ percentile were excluded from the analysis. We also downloaded drug response data across the CCLs (CCL) from the Cancer Therapeutics Response Portal (CTRPv2)^3^. Data coming from CCLs were treated separately according to whether they were derived from solid or liquid tumours. To identify genes whose expression was correlated with drug potency, for each gene and for each drug, we randomly selected 40% of the total number of CCLs (n=672 for solid tumours; n=83 for liquid tumours) and computed the Pearson correlation coefficient (PCC) between the expression of the gene and the effect of the drug expressed in terms of Area Under the Curve (AUC) across the CCLs. This operation was repeated 1,000 times and the mean value of the PCC across the 1000 runs was assigned to the gene/drug pair. The AUC ranges from 0 to 1, with values close to 0 standing for drug sensitivity and those close to 1 for drug resistance. Since the AUC reflects the *in vitro* response of a cell-line to increasing concentrations of the drug, small AUC values are associated with cell sensitivity, while large AUC values, with resistance to the tested drug. Hence, genes with a positive PCC are potential markers of resistance (the more expressed the gene, the higher the concentration needed to inhibit growth), vice-versa, negative PCC values correspond to genes acting as markers of sensitivity. For each drug, we thus generated a drug-gene correlation profile (DGCP) by ranking genes according to their PCC in descending order, so that resistance biomarkers are found at the top of the DGCP, while sensitivity biomarkers at the bottom. In total, we obtained 445 DGCPs, corresponding to 445 drugs, for solid tumours and 414 DGCPs, corresponding to 414 drugs, for liquid tumours. To assess the biological relevance of DGCPs, for each drug with a known mechanistic target, we first selected the Gene Ontology pathways in which the drug target is present from the C5 Molecular Signature Database (MSigDB)^4^ collection; then, for each these pathways, we performed a Gene Set Enrichment Analysis (GSEA) against the drug’s DGCP to assess whether genes in these pathways where significantly enriched at the bottom of drug DGCP, and hence anti-correlated with the AUC of the drug dose-response curve, reasoning that CCLs highly expressing either the target gene or genes involved in the same pathway, should be sensitive to the drug. Our analysis, reported in **Fig. S1C**, confirmed that this is indeed the case.

**Application of DrugSense: computation of the G-score.**

To predict drug sensitivity from a tumour gene expression profile (GEP), DRUGSense applies four distinct Gene Set Enrichment Analysis (GSEA)^5^ for each drug to be tested, as schematised in **Figure 1B**: (I) GSEA against the drug’s DGCP by using as the input set the top most expressed 250 genes in the tumour GEP; (II) as in I, but this time using as input the bottom least expressed 250 genes; (III) GSEA against the tumour’s GEP using as the input the bottom 250 genes of the drug’s DGCP, i.e. the biomarkers of sensitivity; (IV) as in III but this time using as input the top 250 genes of the drug’s DGCP, i.e. the biomarkers of resistance. GSEA was performed using the *fgsea*^6^ package in the R statistical environment version 4.1.2. These four GSEAs result in 4 Enrichments Scores (ES). To compute the p-value associated to each ES for each of the four GSEAs, DrugSense generates a null distribution of ESs by applying GSEA to 10,000 randomised profiles by shuffling 25% of the gene labels of the tumour GEP. Next, a one-tailed test (either left-tailed for strategies I and IV or right-tailed for strategies II and III) was performed to assign a probability value (p-value). This set of four p-values, one for each GSEA, is averaged using the geometric mean to assess overall sensitivity of the drug (i.e., the G-score). As the four GSEA analyses may be potentially correlated, integration p-values using geometric mean may not be statistically appropriate. We therefore also applied the Brown’s method for p-value integration, which specifically accounts for correlations, as an alternative approach. Despite differences in the absolute values of the resulting p-values, we observed that the positive predicted values were identical for both methods, as shown in **the Supplementarty Figure S3A**. This happens as the ranked list obtained by sorting according to the Brown’s p-values are nearly identical to those obtained when sorting according to geometric mean of p-values.

**Validation of DrugSense on cancer cell lines (CCLs).**

Precision of DrugSense in predicting drug sensitivity was evaluated in CCLs using two publicly available datasets of bulk gene expression profiles of untreated cells: (1) RNA-sequencing data (v21Q4) of 1,375 CCLs downloaded from the CCLE database, of which 775 in common with the drug response CTRPv2 dataset (672 solid + 83 liquid CCLs); (2) microarray expression profiles from the GDSC database (E-MTAB-3610), which includes of 1,011 CCLs, of which 574 in common with the CTRPv2 dataset (475 solid + 99 liquid CCLs). To determine if a cell line was sensitive or not to a specific drug, we exploited the CTRPv2 drug response dataset, which includes drug response in terms of Area Under the Curve (AUC) of the dose-response of CCLs. Drug potency across solid and liquid CCLs were kept apart and analysed separately. Specifically, for each drug we converted the distribution of AUC values across CCLs to Z-scores and then defined a CCL sensitive to the drug if the corresponding Z-score was in the 5^th^ percentile. The gold standard for the CCLE dataset was built by assigning to each of 672 × 445 (=299,040) and 83 x 414 (=34,362) cell line/drug pairs the value of 1, if the CCL is sensitive to the drug, and 0 otherwise. Analogously, the gold standard for the GDSC dataset consists of the 475 x 445 (=211,375) and 99 x 414 (=40,986) CCL/drug pairs. CCL/drug pairs in the gold standards were then ordered according to the G-scores computed by DrugSense on the expression profiles. Finally, the Positive Predicted Values (PPV= TP/ (TP + FP) was computed by binning cell line/drug pairs in percentiles. The PPVs were normalized against the PPV obtained from a random ordering of cell line/drug pairs.

**Validation of DrugSense on PDX data**

Precision of DrugSense in predicting drug sensitivity was evaluated in PDX mouse models following the same pipeline as described above for CCLs. We downloaded from the Childhood Cancer Therapeutics (PCAT)^7^ data portal, the drug response data of 39 PDX models treated with one or more drugs for which a DGCP was available in DrugSense. We then downloaded bulk gene expression profiles (RNA-seq) of these 39 PDX models from the Paediatric Preclinical Testing Consortium (PPTC)^8^. Drug responses in PCAT are categorized into six levels, hence the gold standard was built by assigning to each PDX/drug pair the value of 1 if the PDX responded to the drug (either with a maintained complete, complete, or partial response) and 0 otherwise.

**Benchmarking of DrugSense in the NCI DREAM Challenge**

To benchmark DrugSense's ability to predict drug response against other previously published approaches, we used the data provided by the NCI-DREAM drug prediction challenge^9^. The challenge aimed to rank 18 breast cancer cell lines from the most sensitive to the most resistant for each of 28 compounds. We thus generated Drug Gene Correlation Profiles (DGCPs) only for the 4 out of 28 compounds that were present in the collection of compounds that were used to train DrugSense (445 + 306 retrieved from GDSC). DrugSense's predictions were evaluated using RNA-seq data from 16 cell-lines, including those provided by the NCI-DREAM challenge (10) and those collected from the Gene Expression Omnibus (GEO) repository (6; ACNO: GSE48213). We then computed the performance indices as described in the NCI-DREAM challenge^9^ (i.e. the single-drug and overall weighted, probabilistic c-index (wpc-index)). We compared DrugSense's performance with that of the nine other drug sensitivity prediction algorithms present in the NCI-DREAM challenge that used the same training data, i.e. gene expression (e), RNA-seq (n), and outside information (OI).

**Application of DrugSense to HB.**

Microarray-based gene expression data of 25 hepatoblastoma (HB) samples from Affymetrix HG-U133A 2.0 GeneChip™, reported in *Cairo et al.*^10^, were first normalized using robust multi-array average method (R package affy, v1.72.0)^11^ and aligned with the cell line expression profiles of the GDSC dataset. To this end, we combined the HB microarrays and GDSC microarrays by keeping only probes with the best reported match (U133PlusVsU219_BestMatch.zip in the Affymetrix's website). The combined microarrays were re-normalized applying quantile normalization (R package *limma*, v3.50.0)^12^. Then, we applied the function *ComBat* from the R/Bioconductor package *sva*^13^ to eliminate batch effects between the two platforms. Finally, probe to gene conversion was achieved by keeping, among the probes associated with the same gene, only the one with the highest Median Absolute Deviations (MAD) value across all samples. We then applied DrugSense to these processed profiles comprising 25 HB samples to predict the potency of each drug in each sample. Drugs were ranked by G-score in ascending order according to their predicted potency and individual rankings related to either the C1 or the C2 samples were aggregated using the *RankAggreg* function from the homonymous R package^14^, using default parameters.

**Cell culture**

Seven PDX cell lines (PDX214, PDX243, PDX282^15^, PDX295, PDX303, PDX344, and PDX346)^16^ were kindly donated by Stefano Cairo (XenTech, Evry, France) and maintained in Advanced DMEM/F12 (Life Technologies, Carlsbad, CA, USA) supplemented with 10% (v/v) fetal bovine serum (FBS), 1% (v/v), 1% L-Glutamine (v/v) (Thermo Fisher, Waltham, MA, USA) and the ROCK inhibitor Y-27632 (Selleckchem, Chesterbrook, PA, USA) in a final concentration of 20 µM. Two dermal fibroblast cell lines adult HDFa and neonatal HDFn (ATCC) were also included as non-cancerous controls and incubated in RPMI 1640 (Life Technologies) supplemented with 10% (v/v) fetal bovine serum (Life Technologies) and 1% (v/v) penicillin-streptomycin (Invitrogen). All cell lines were kept at 37 ºC in a humidified atmosphere containing 5% CO2 and universal mycoplasma detection kit (LGC Standards, Teddington, UK) was used for regular mycoplasma testing.

**Viability assay**

MTT (3-(4, 5-dimethylthiazol-2-yl)-2, 5-diphenyltetrazolium bromide) (Sigma-Aldrich, St. Louis MO, USA) viability assay was performed to determine drug responses of the cells. 5x10^4^ cells/well were seeded in 96 well plate 24h prior to drug exposure. All cell lines were exposed to 10 increasing concentrations of dinaciclib or alvocidib (Selleckchem) ranging from 0.005 µM to 100 µM concentrations in 1:3 serial dilutions. Absorbance values were measured at the Sunrise plate reader (Tecan Group. Männedorf, Switzerland) after 48h incubation, and area under the drug response curves were calculated using GraphPad Prism 7 (La Jolla, CA, USA).

**Apoptosis assay**

Apoptotic cell count was determined via CellEvent™ Caspase-3/7 Green Detection Reagent (Thermo Fisher) according to manufacturer`s instructions. 2x10^5^ cells/well were seeded in 24-well plate 24h prior to drug exposure. Following 24h dinaciclib (0.1 µM) or alvocidib (0.1 µM) exposure, active forms of Caspase 3/7 substrate were observed in the GFP channel of the EVOS M700 cell imaging system (Thermo Fisher). Counting of the apoptotic cells was carried out by the EVOS analysis software.

**Proliferation assay**

Click-iT™ EdU Cell Proliferation Kit (Thermo Fisher) was applied according to manufacturer’s instructions for the detection of proliferating cell portions. 2x10^5^ cells/well seeded in 24-well plate. Next day, the cells were labelled with 100 µM ethynyl deoxyuridine (EdU) and exposed to dinaciclib (0.1 µM) or alvocidib (0.1 µM) for 24h. Following 3.7% paraformaldehyde fixation and Triton-X permeabilization, cells were stained with Alexa Fluor^®^ 555 azide (RFP channel) for 30 mins. Hoechst 33342 (DAPI channel) was used for staining of nuclei. Imaging and the analysis of the assay was performed at the EVOS M7000 system (Thermo Fisher).

**Western blot analysis**

Whole cell lysates were extracted from 3 × 10^7^ cells grown in 100 mm cell culture petri dishes for 24h. Cell pellets were incubated in cell lysis buffer (30 mM Tris/HCl, pH 8.0, 150 mM NaCl, 1% Triton-X, 10% glycerol, 1X Complete protease inhibitor tablet (Sigma-Aldrich)) with the addition of 100 mM phenylmethylsulfonylfluorid (PMSF), 1 M Dithiothreitol (DTT), 200 mM sodium orthovanadate (Na2VO4), 500 mM sodium fluoride (NaF), and 100 mM β-glycerophosphate. Then, lysates were centrifuged at 13,000 rpm for 30 min at 4°C. Protein concentrations were determined by Bradford assay (Bio-Rad, Hercules, CA, USA), and 20 µg whole cell lysates were separated via SDS-gel electrophoresis, using Novex™ WedgeWell™ 8% pre-cast tris-glycine gels (Thermo Fisher). Transfer of the proteins were done via Turbo Blot (Bio-Rad) with the help of Trans-Blot Turbo Mini 0.2 µm Nitrocellulose Transfer Packs (Bio-Rad). The membrane was incubated overnight at 4^o^C in anti-CDK9 antibody (Sigma Aldrich, #HPA006738) diluted 1:1,000. Anti-beta-actin in a 1:10.000 dilution (Cell Signaling Technologies, Danver, Ma, USA, #4967S) served as a loading control. Following the incubation with a secondary goat-anti-rabbit HRP antibody (Dako Denmark, Glostrup, Denmark, # P0448) in a 1:5,000 dilution, proteins were detected by enhanced chemo-luminescence (ECL) reagent (Amersham Biosciences, Amersham, UK) using the ChemiDoc™ XRS+ imaging system (Bio-Rad). All antibody dilutions were prepared in 5% milk in PBS-T.

**In vivo study**

Animal studies were carried out by XenTech according to previous studies^15,17^. PDX282 tumours were implanted into Athymic nude-Foxn1nu mice. After latency period, mice with subcutaneously growing tumours between 75 and 288 mm^3^ were allocated to each treatment arm as 7 mice/group, according to their tumour volume to maintain homogenous mean and median tumour volume in each arm. Mice were treated with vehicle, alvocidib (5 mg/kg) or dinaciclib (20 mg/kg) by intraperitoneal (i.p.) injection for 5 subsequent days per week. Tumour volume (TV) was evaluated by measuring tumour diameters with a calliper, 2 or 3 times a week during latency and treatment period. The formula TV (mm^3^) = [length (mm) x width (mm)^2^]/2 was used, where the length and the width are the longest and the shortest diameters of the tumour. All animals were weighed at tumour measurement time points and visually inspected every day for physical appearance, behaviour, and clinical changes. Animals that reached to maximum TV were sacrificed.

**Immunohistochemistry**

Four-micrometer-thick tissue sections from paraffin blocks were baked for 20 min at 65 ^o^C. Antigen retrieval was performed with a PT Link instrument (Agilent), using Citrate buffer (97^o^C, 20 min). Sections were immersed in H_2_O_2_ aqueous solution (Blocking peroxidase reagent, Agilent) for 10 min to exhaust endogenous peroxidase activity and then covered with 1% blocking reagent (Roche, Mannheim, Germany) in PBS, to block nonspecific binding sites. Sections were then incubated with primary antibody, CDK9 (Sigma-Aldrich catalog numner HPA006738), 1/300, overnight, at 4^o^C, in a humid chamber. Later, rabbit linker (Agilent) and HRP polymer conjugated secondary antibody (Visualization reagent, Agilent) were applied for 15 min and 1h, respectively, at room temperature in a humid chamber. Afterwards, 3,3-diaminobenzidine were applied for 5 min to develop immunoreactivity. Slides were then counterstained with haematoxylin and mounted in DPX mountant. Images from immunostainings were taken using Primo Star LED microscope (Zeiss) alongside ZEISS ZEN Imaging software. Percentage of tumour-stained area was calculated by examination of 3-6 random high-power fields (x40) and quantified with specific thresholds using ImageJ software v.45.s (National Institutes of Health).

## NanoString nCounter

To classify tumors according to the C1/C2 classification, 38 out of our 46 HBs (for 8 tumours, no additional tissue was available) were profiled using the NanoString nCounter Technology with a manually curated list of 18 genes, including HB markers and genes of key signalling pathways (**Supplementary Table 3**). Here, haematoxylin and eosin (H&E)-stained slides were used to select viable tumour areas by an expert pathologist. RNA from tumour FFPE unstained sections was extracted using truXTRAC FFPE RNA microTUBE purification Kits (COVARIS, Woburn, MA). RNA quality control was assessed using Tape Station RNA chips (Agilent) and RNA quality was measured using DV200 metrics. RHOT2 and PNN genes were used as a housekeeping genes as already reported^18^. NanoString output files were analyzed by nSolver Analysis Software 4.0.

**REFERENCES**

1. Barretina, J. *et al.* The Cancer Cell Line Encyclopedia enables predictive modelling of anticancer drug sensitivity. *Nature* **483**, 603–607 (2012).

2. Robinson, M. D., McCarthy, D. J. & Smyth, G. K. edgeR: a Bioconductor package for differential expression analysis of digital gene expression data. *Bioinformatics* **26**, 139–140 (2010).

3. Seashore-Ludlow, B. *et al.* Harnessing Connectivity in a Large-Scale Small-Molecule Sensitivity Dataset. *Cancer Discovery* **5**, 1210–1223 (2015).

4. Liberzon, A. *et al.* Molecular signatures database (MSigDB) 3.0. *Bioinformatics* **27**, 1739–1740 (2011).

5. Subramanian, A. *et al.* Gene set enrichment analysis: A knowledge-based approach for interpreting genome-wide expression profiles. *Proc. Natl. Acad. Sci. U.S.A.* **102**, 15545–15550 (2005).

6. Korotkevich, G. *et al.* *Fast gene set enrichment analysis*. http://biorxiv.org/lookup/doi/10.1101/060012 (2016) doi:10.1101/060012.

7. Yang, J. *et al.* PCAT: an integrated portal for genomic and preclinical testing data of pediatric cancer patient-derived xenograft models. *Nucleic Acids Research* **49**, D1321–D1327 (2021).

8. Rokita, J. L. *et al.* Genomic Profiling of Childhood Tumor Patient-Derived Xenograft Models to Enable Rational Clinical Trial Design. *Cell Reports* **29**, 1675-1689.e9 (2019).

9. Costello, J. C. *et al.* A community effort to assess and improve drug sensitivity prediction algorithms. *Nat Biotechnol* **32**, 1202–1212 (2014).

10. Cairo, S. *et al.* Hepatic Stem-like Phenotype and Interplay of Wnt/β-Catenin and Myc Signaling in Aggressive Childhood Liver Cancer. *Cancer Cell* **14**, 471–484 (2008).

11. Gautier, L., Cope, L., Bolstad, B. M. & Irizarry, R. A. affy--analysis of Affymetrix GeneChip data at the probe level. *Bioinformatics* **20**, 307–315 (2004).

12. Ritchie, M. E. *et al.* limma powers differential expression analyses for RNA-sequencing and microarray studies. *Nucleic Acids Research* **43**, e47–e47 (2015).

13. Leek, J. T., Johnson, W. E., Parker, H. S., Jaffe, A. E. & Storey, J. D. The sva package for removing batch effects and other unwanted variation in high-throughput experiments. *Bioinformatics* **28**, 882–883 (2012).

14. Pihur, V., Datta, S. & Datta, S. RankAggreg, an R package for weighted rank aggregation. *BMC Bioinformatics* **10**, 62 (2009).

15. Nicolle, D. *et al.* Patient-derived mouse xenografts from pediatric liver cancer predict tumor recurrence and advise clinical management. *Hepatology* **64**, 1121–1135 (2016).

16. Eloranta, K. *et al.* Chloroquine Triggers Cell Death and Inhibits PARPs in Cell Models of Aggressive Hepatoblastoma. *Front Oncol* **10**, 1138 (2020).

17. Xue, Y. & Mansoori, G. A. Self-assembly of diamondoid molecules and derivatives (MD simulations and DFT calculations). *Int J Mol Sci* **11**, 288–303 (2010).

18. Carrillo-Reixach, J. *et al.* Epigenetic footprint enables molecular risk stratification of hepatoblastoma with clinical implications. *J Hepatol* **73**, 328–341 (2020).
